# PKM2-mediated collagen XVII expression is critical for wound repair

**DOI:** 10.1172/jci.insight.184457

**Published:** 2025-01-21

**Authors:** Yangdan Liu, Chiakang Ho, Dongsheng Wen, Jiaming Sun, Yuxin Liu, Qingfeng Li, Yifan Zhang, Ya Gao

**Affiliations:** Department of Plastic and Reconstructive Surgery, Shanghai Ninth People’s Hospital, School of Medicine, Shanghai Jiao Tong University, Shanghai, China.

**Keywords:** Dermatology, Therapeutics, Collagens

## Abstract

Chronic wounds have emerged as a tough clinical challenge. An improved understanding of wound-healing mechanisms is paramount. Collagen XVII (COL17), a pivotal constituent of hemidesmosomes, holds considerable promise for regulating epidermal cell adhesion to the basement membrane as well as for epidermal cell motility and self-renewal of epidermal stem cells. However, the precise role of COL17 in wound repair remains elusive, and the upstream regulatory mechanisms involved have not been fully elucidated. In this study, we delineated the temporal and spatial expression patterns of COL17 at the epidermal wound edge. Subsequently, we investigated the indispensable role of COL17 in keratinocyte activation and reepithelialization during wound healing, demonstrating the restoration of the normal repair process by COL17 overexpression in diabetic wounds. Notably, we identified a key transcriptional signaling pathway for COL17, wherein pyruvate kinase isozyme M2 (PKM2) promotes phosphorylation of STAT3, leading to its activation and subsequent induction of COL17 expression upon injury. Ultimately, by manipulating this pathway using the PKM2 nuclear translocator SAICAR, we revealed a promising therapeutic strategy for enhancing the healing of chronic wounds.

## Abstract 2

Collagen XVII is up-regulated in a PKM2-mediated manner, which enhances the repair of chronic wounds.

## Graphical abstract

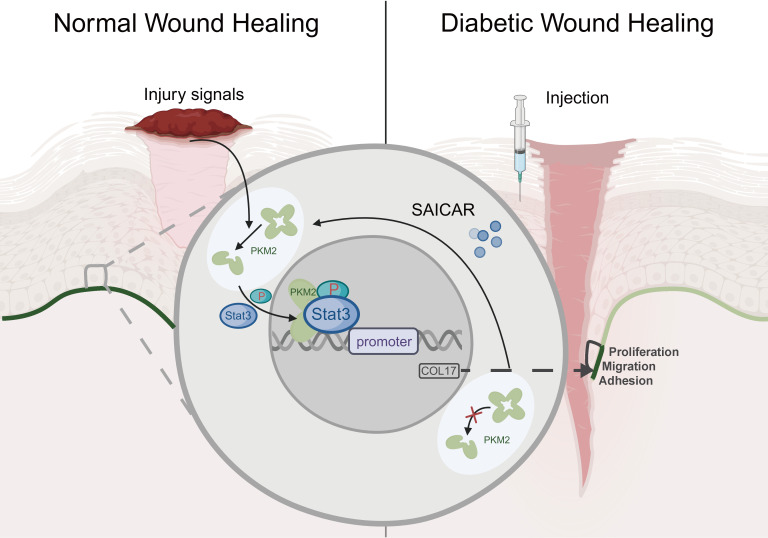

## Introduction

Upon tissue damage, the skin is particularly exposed to injuries that necessitate rapid repair. However, the failure to heal is an intractable clinical problem that results in chronic wounds. Chronic wounds have long been considered a substantial clinical, social, and economic challenge, imposing an enormous burden on patients and society (1). Understanding the intrinsic molecular mechanisms that regulate the wound-healing process may reveal targets for more effective therapy for chronic wounds. Epithelial dynamics during wound healing are among the keys to effective healing (2). Migration is typically observed at the leading edge of the wound, while proliferation occurs at some distance away from the edge, which leads to the establishment of two continuous compartments and promotes reepithelialization (3). Although the key steps of wound healing are well described at the tissue level, many unanswered questions remain regarding the precise molecular and cellular mechanisms underlying these phenomena.

Collagen XVII (COL17) is a transmembrane protein that is mainly expressed by basal keratinocytes. It serves as a structural component of hemidesmosomes in the dermal-epidermal basement membrane zone and plays an important role in keratinocyte physiology (4). The roles of COL17 in the process of wound healing have been further elucidated in recent years. A robust increase in COL17 has been observed at the wound edge, and wound closure is hampered in COL17-KO mice (5, 6). Cells with high expression of COL17 demonstrate a heightened capacity for cell renewal over differentiation, which helps compensate for lost cells during reepithelialization (7). COL17 also influences the velocity and direction of migration of keratinocytes (8, 9). Thus, COL17 is a promising therapeutic target for wound repair. Nevertheless, the mechanisms that induce the extensive expression and distribution of COL17 at the wound edge remain elusive. The absence of sufficient investigations into the upstream mechanisms creates challenges for screening COL17-inducing drugs.

Therefore, in the present study, we examined the expression profile of COL17 and performed functional validation to substantiate its role in wound healing both in vitro and in vivo. Subsequently, we identified a regulatory pathway involving nuclear PKM2, which promoted STAT3 phosphorylation and induced its nuclear translocation, thereby facilitating COL17 expression after injury. Modulation of PKM2 nuclear translocation enhanced keratinocyte functions and expedited reepithelialization, suggesting a promising therapeutic strategy for impaired wounds.

## Results

### Expression pattern of COL17 in cutaneous wound healing.

**Figure 1:**
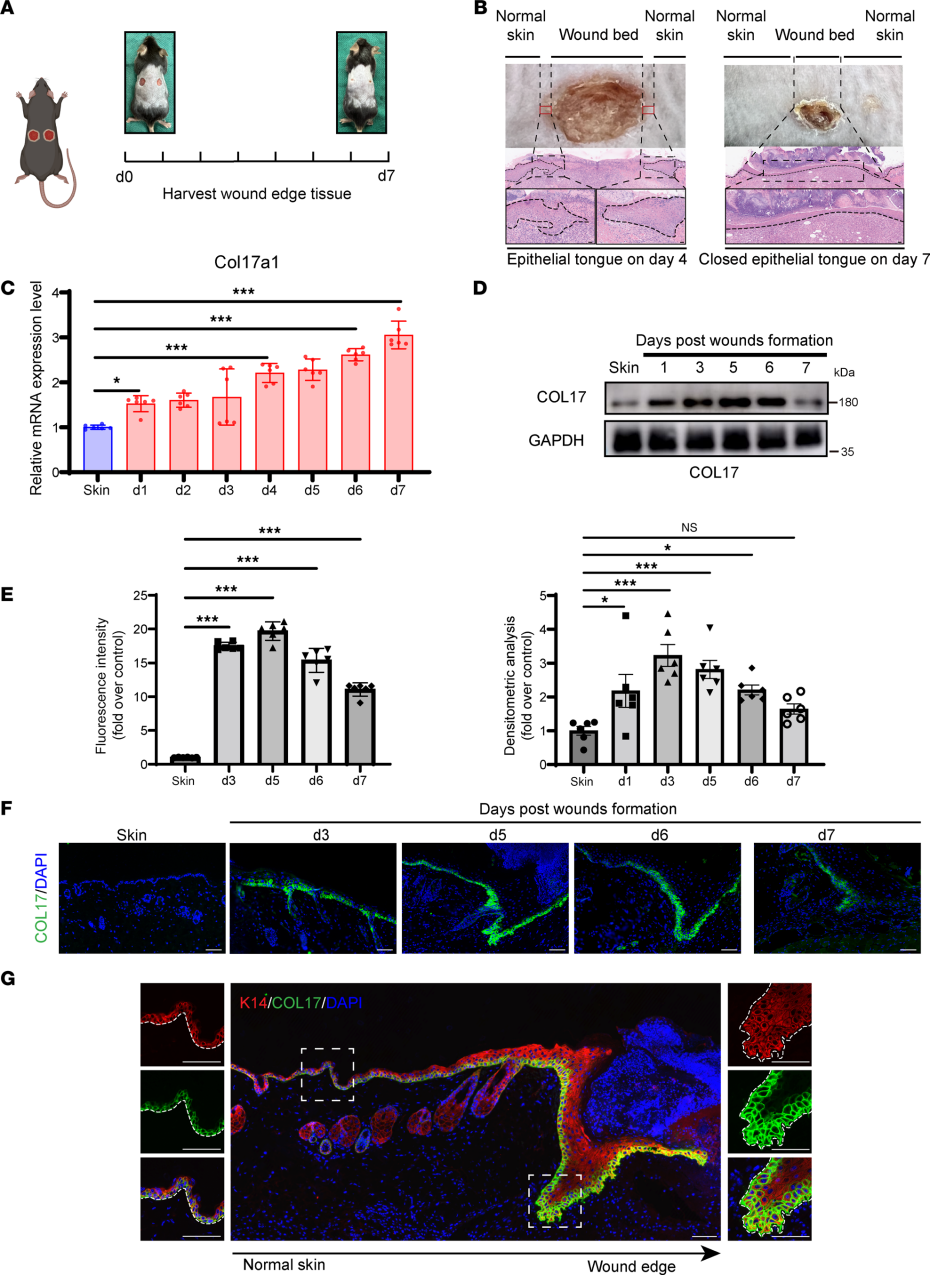
Expression of COL17 during wound repair. (**A**) Schematic representation of the murine tissue harvesting strategy. (**B**) Representative images of wounds and photomicrographs of H&E-stained wound edge sections on PWD 4 and 7. Scale bar: 20 μm. (**C**) Relative mRNA expression level of Col17a1 in wound edge tissues on PWD 0–7. (**D**) Protein expression level of COL17 in wound edge tissues on PWD 0–7. (**E**) Quantification analysis of fluorescence intensity of **F**. (**F**) Representative immunofluorescence images of the wound edge showing COL17 (green) on PWD 0–6 (scale bar: 20 μm). (**G**) Representative immunofluorescence images of the wound edge showing COL17 (green) and K14 (red) on PWD 5 (scale bar: 20 μm). The data are presented as the mean ± SD (*n* = 6 or 9 independent animals). *P < 0.05, ***P < 0.001. ANOVA was used to compare multiple groups.

Recent studies have shown that dermal-epidermal junctions play a key role in the regulation of skin wound repair (10, 11). Considering the integral role of COL17 within the hemidesmosome, a crucial structure of the dermal-epidermal junction, we investigated its expression at wound margins after injury. Wound margin tissues were collected from full-thickness biopsy wounds in C57BL/6 mice from postwound day (PWD) 0 to 7, corresponding to the closure of the epithelial tongue (Figure 1, A and B). From PWD 1 to 5, both the mRNA and protein levels of COL17 were significantly increased. However, while COL17 mRNA expression remained elevated until PWD 7, COL17 protein levels began to decrease after PWD 5, reaching near-baseline levels by PWD 7 (Figure 1, C and D). These findings suggest that COL17 transcription remained consistently active throughout the repair process, while the protein expression level may have been influenced by some unknown mechanisms at later stages. To delineate the role of COL17 across different skin layers, we further characterized its temporal and spatial expression at wound margins using immunofluorescence staining. As anticipated, COL17 was transiently induced in basal epithelial cell membranes following injury (Figure 1, E and F). Notably, its expression gradually decreased with increasing distance from the wound bed (Figure 1G). Taken together, these spatial expression patterns of epidermal COL17 support its potential regulatory involvement in cutaneous wound healing.

### COL17 is required for effective regulation of wound reepithelialization.

**Figure 2:**
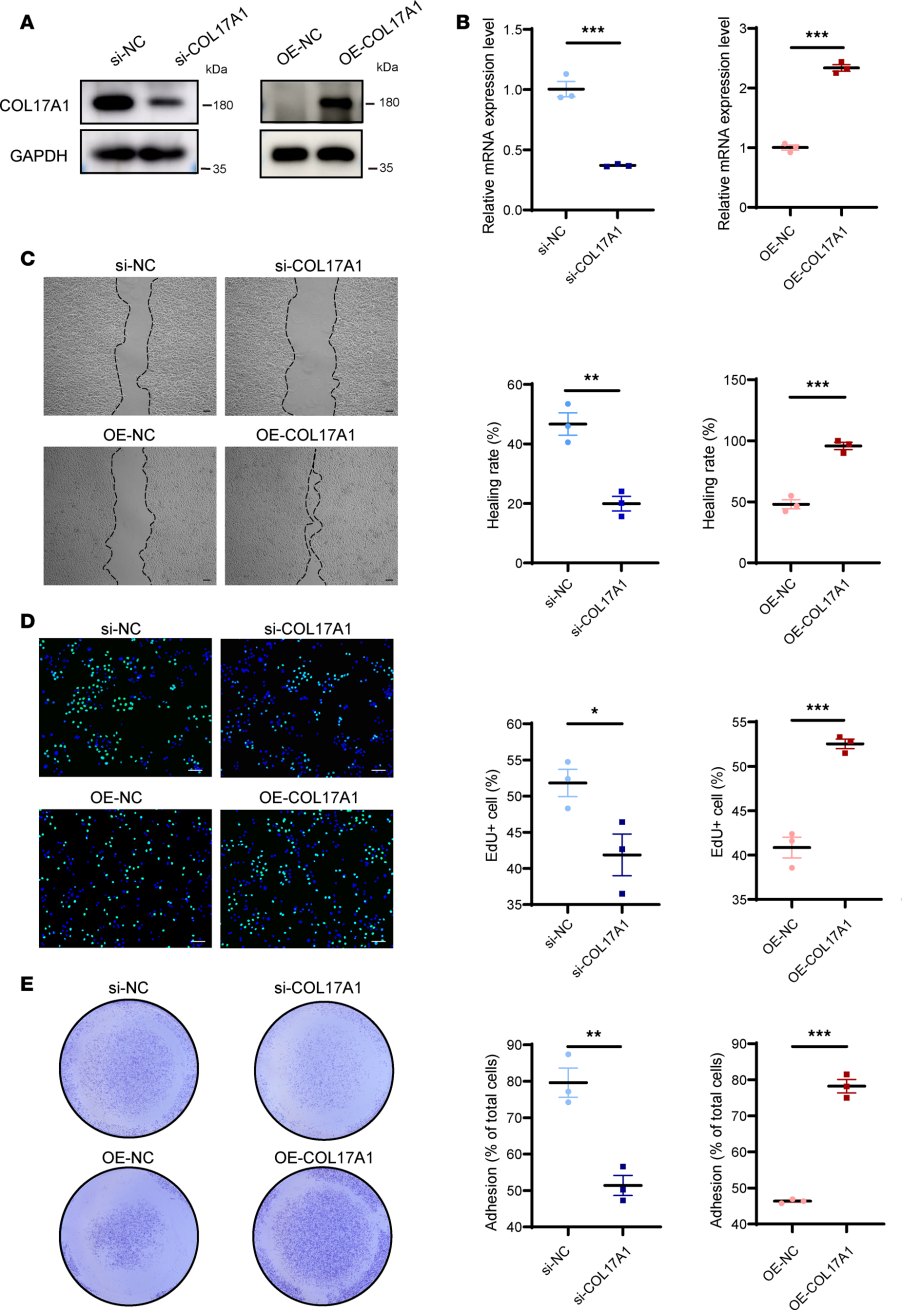
COL17 promotes keratinocyte migration, proliferation, and adhesion. (**A**) Protein expression level of COL17 in HaCaT cells transfected with siRNA or COL17A1 overexpression plasmids. (**B**) Relative mRNA expression level of COL17A1 in HaCaT cells transfected with siRNAs or COL17A1 overexpression plasmids. (**C**) Wound-healing assay and quantification analysis of HaCaT cells with COL17 knockdown or overexpression (scale bar: 100 μm). (**D**) EdU (green) proliferation assay and quantification analysis of HaCaT cells with COL17 knockdown or overexpression (scale bar: 100 μm). (**E**) Adhesion assay and quantification analysis of HaCaT cells with COL17 knockdown or overexpression. The data are presented as the mean ± SEM (*n* = 3 independent experiments). *P < 0.05, **P < 0.01, ***P < 0.001. Independent-sample *t* test was used for comparisons between the 2 groups.

Following injury, epithelial cells undergo reepithelialization by adhering, proliferating, and migrating. Previous studies have shown delayed wound closure in mice lacking COL17 (5, 6), indicating its potential role in this process. Here, we further investigated whether the absence or overexpression of COL17 in keratinocytes affected cell migration, proliferation, and adhesion (Figure 2, A and B). Our findings revealed that treatment with COL17A1 siRNA significantly decreased the wound-healing rate and the percentage of proliferating and adherent cells, whereas treatment with COL17A1 expression plasmids increased these parameters, suggesting the importance of COL17 in keratinocyte activation (Figure 2, C–E).

**Figure 3:**
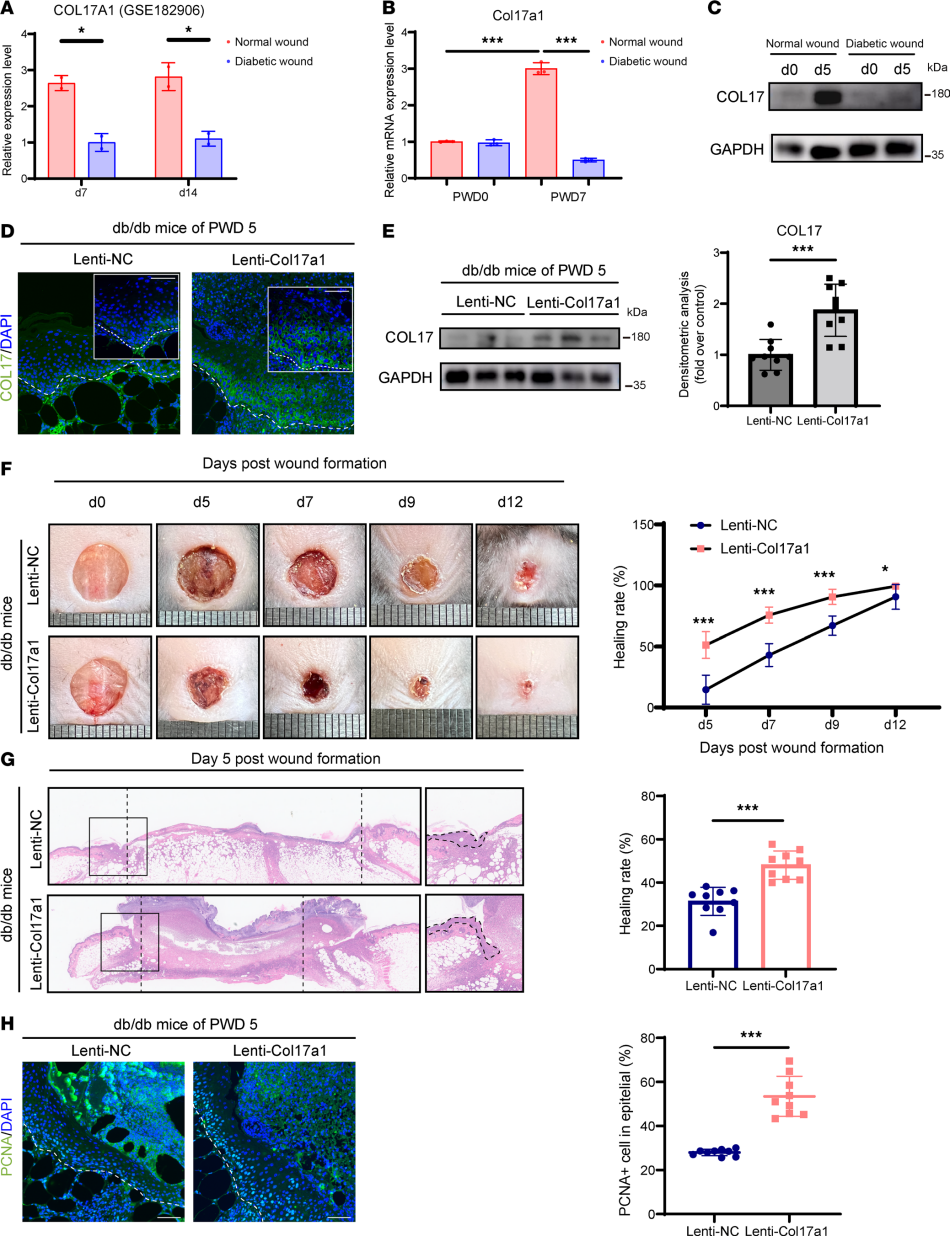
Overexpression of COL17 ameliorates impaired wound healing in db/db mice. (**A**) Analysis of Col17a1 mRNA expression (data from GSE182906) in normal and diabetic wound edge tissues on PWD 7 and 14. (**B**) Relative mRNA expression level of Col17a1 in normal and diabetic wound edge tissues on PWD 0 and 7. (**C**) Protein expression level of COL17 in normal and diabetic wound edge tissues on PWD 0 and 5. (**D**) Representative immunofluorescence images of COL17 (green) in the wounds of negative control (NC) or Col17a1 lentivirus-transfected db/db mice at PWD 5 (scale bar: 20 μm). (**E**) Western blot and quantification analysis of COL17 at wound edges on PWD 5 in db/db mice treated with NC or Col17a1 lentivirus. (**F**) Representative chronological images of wounds and analysis of the wound area healing rate in NC or Col17a1 lentivirus-transfected db/db mice. (**G**) Representative H&E images of wounds and analysis of the wound gap healing rate on PWD 5 in NC or Col17a1 lentivirus-transfected db/db mice. (**H**) Representative immunofluorescence images of PCNA (green) and the percentage of PCNA-positive cells in the wound edges of NC or Col17a1 lentivirus-transfected db/db mice on PWD 5 (scale bar: 20 μm). The data are presented as the mean ± SD (*n* = 9 independent animals). *P < 0.05, ***P < 0.001. Independent-sample *t test* was used for comparisons between the 2 groups and ANOVA with Tukey’s test was used to compare multiple groups.

In addition to its role in normal wound healing, the involvement of COL17 in chronic wounds was assessed. Analysis of single-cell RNA-sequencing data from GEO GSE199939 has shown that COL17 expression was significantly reduced in chronic wounds of patients with diabetes (Supplemental Figure 10; supplemental material available online with this article; https://doi.org/10.1172/jci.insight.184457DS1). Both reanalysis of a previous dataset (GEO GSE182906) and our quantitative real-time PCR (RT-qPCR) results confirmed a significant reduction in COL17 mRNA levels in murine delayed healing wounds on PWD 7 and 14 (Figure 3, A and B). Western blotting revealed that COL17 protein levels were markedly decreased in murine delayed healing wounds on PWD 5 (Figure 3C). These findings suggest that dysregulated COL17 expression contributed to defective healing.

Given the deficiency of COL17 in diabetic wounds, we investigated whether topical overexpression of COL17 in diabetic mice could enhance wound healing. To explore this possibility, we administered lentiviruses around the wound edge and confirmed that Col17a1 was overexpressed in the skin (Figure 3, D and E, and Supplemental Figure 9). Our results demonstrated accelerated wound healing and reepithelialization in diabetic mice overexpressing Col17a1 (Figure 3, F and G). Moreover, the percentage of PCNA-positive cells was increased in the lenti-Col17a1 group, indicating that COL17 promotes the proliferation of epithelial cells at wound edges (Figure 3H). These findings collectively suggest that altered levels of epidermal COL17 impacted both normal reepithelialization and the chronic wound phenotype.

### Col17a1 is transcriptionally activated by direct binding of STAT3 during wound healing.

**Figure 4:**
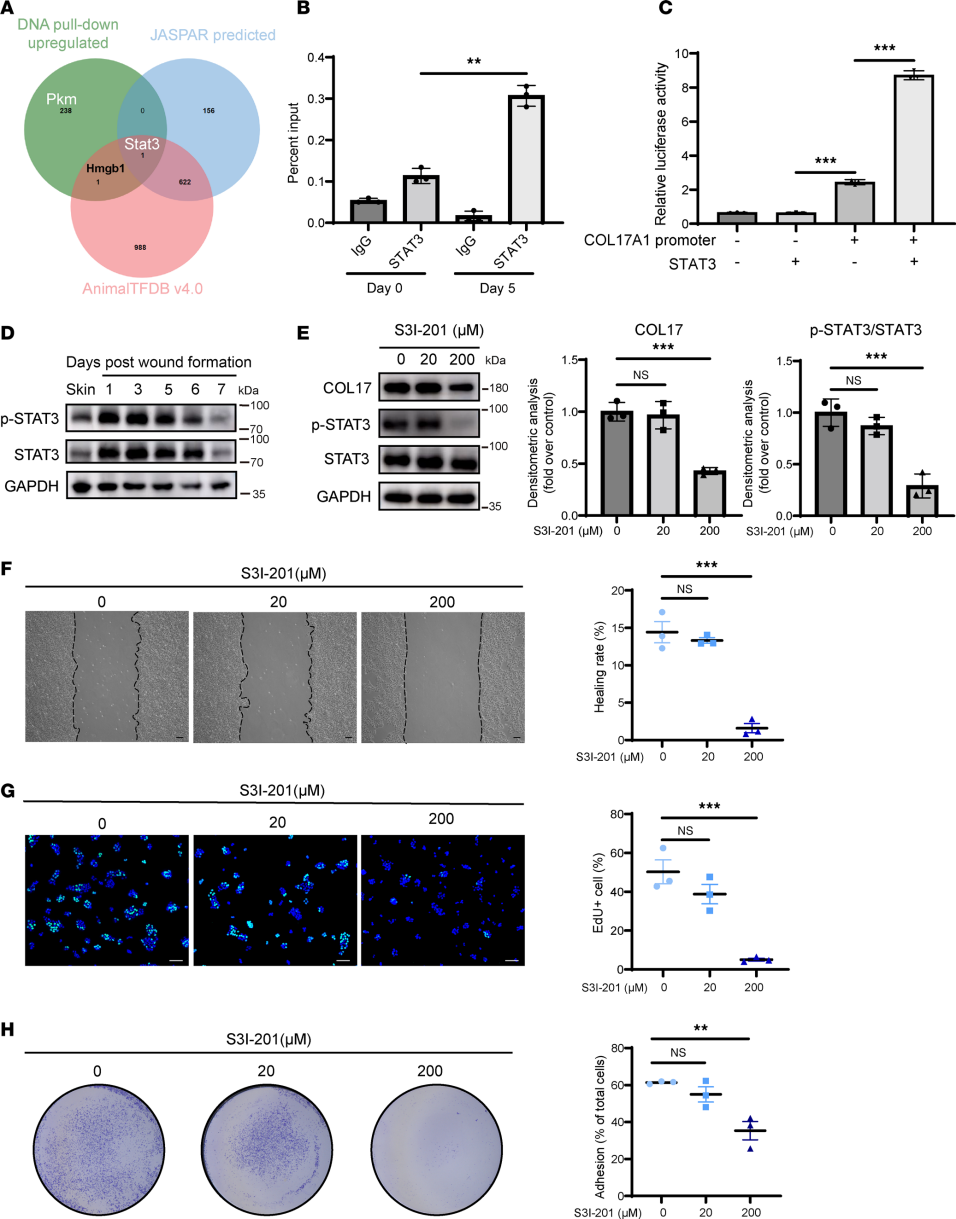
STAT3 promotes COL17A1 transcription and keratinocyte activation. (**A**) DNA pull-down plus LC-MS/MS upregulated protein assay results with AnimalTFDB v4.0 data and JASPAR prediction. (**B**) ChIP analysis of STAT3 enrichment at the Col17a1 promoter in murine wound edge tissues on PWD 0 and 5. (**C**) Luciferase reporter assay of COL17 transcriptional activity with or without STAT3 transfection. (**D**) Chronological protein expression levels of phosphorylated and total STAT3 in wound edge tissues from PWD 0 to 7. (**E**) Western blot and quantification of COL17 and phosphorylated and total STAT3 in HaCaT cells treated with different concentrations of S3I-201. (**F**) Wound-healing assay and quantification analysis of HaCaT cells treated with different concentrations of S3I-201 (scale bar: 100 μm). (**G**) EdU (green) proliferation assay and quantification analysis of HaCaT cells treated with different concentrations of S3I-201 (scale bar: 100 μm). (**H**) Adhesion assay and quantification analysis of HaCaT cells treated with different concentrations of S3I-201. The in vitro data are presented as the mean ± SEM (*n* = 3 independent experiments). **P < 0.01, ***P < 0.001. ANOVA with Tukey’s test was used to compare multiple groups.

Given the critical role of active COL17 transcription in wound healing, we investigated the upstream regulation of COL17 to identify potential targets for intervention in chronic wounds. Initially, we conducted high-throughput screening to identify transcription factors (TFs) that bind to the COL17 promoter (Figure 4A). Using DNA probes designed for the COL17 promoter, protein pull-down assays were conducted on wounds from PWD 0 and PWD 5. Analysis by liquid chromatography tandem mass spectrometry (LC-MS/MS) revealed a total of 240 proteins that were identified, with a greater enrichment of unique peptides in PWD 5 wounds than in PWD 0 wounds. Among these proteins, only 2, STAT3 and HMGB1, are known TFs or TF cofactors (AnimalTFDB v4.0; https://guolab.wchscu.cn/AnimalTFDB4/#/). Further prediction using JASPAR identified STAT3 as the most likely TF regulating COL17 transcription. To verify the direct binding of STAT3 to the COL17 promoter, we performed ChIP-qPCR (Figure 4B). In wounds from PWD 5, the predicted binding sequence of the COL17 promoter showed robust enrichment of STAT3 binding compared with that of the IgG controls, indicating that COL17 is a direct target of STAT3. To assess whether STAT3 transactivates COL17 gene expression, we generated reporter gene constructs by subcloning the COL17 promoter (–1,920 kb to +80 kb) into the pGL3-luciferase reporter vector. The luciferase activity of the reporter gene significantly increased with STAT3 overexpression (Figure 4C). These findings collectively demonstrate that STAT3 transcriptionally activated COL17 upon wounding through direct binding.

**Figure 5:**
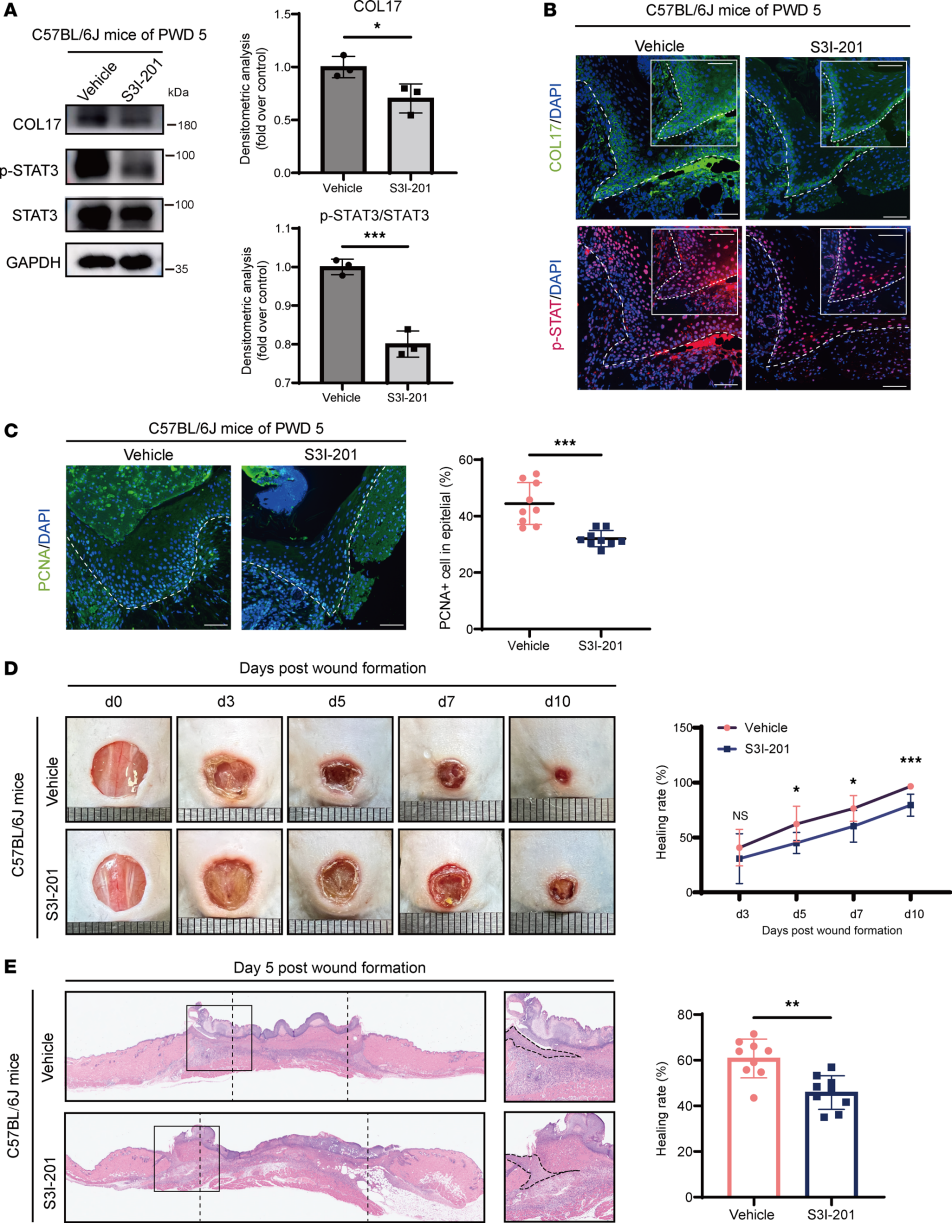
Inhibition of STAT3 activation suppresses wound healing. (**A**) Western blot analysis of COL17 and phosphorylated and total STAT3 in C57BL/6J murine PWD 5 wound edges treated with vehicle or S3I-201. (**B**) Representative immunofluorescence images of COL17 (green) and p-STAT3 (red) expression in wound edges on PWD 5 in C57BL/6J mice treated with vehicle or S3I-201 (scale bar: 20 μm). (**C**) Representative immunofluorescence images of PCNA (green) and the percentage of PCNA-positive cells at the wound edges on PWD 5 in C57BL/6J mice treated with vehicle or S3I-201 (scale bar: 20 μm). (**D**) Representative chronological images of wounds and analysis of the wound area healing rate in mice treated with vehicle or S3I-201. (**E**) Representative H&E images of wounds and analysis of the wound gap healing rate on PWD 5 in mice treated with vehicle or S3I-201. The in vivo data are presented as the mean ± SD (*n* = 9 independent animals). *P < 0.05, **P < 0.01, ***P < 0.001. Independent-sample *t* test was used for comparisons between the 2 groups and ANOVA with Tukey’s test was used to compare multiple groups.

Phosphorylation of STAT3 contributes to its nuclear translocation and transcriptional regulation capacity (12). Our results revealed that phosphorylated STAT3 levels transiently increased during wound healing, paralleling the changes in the expression of COL17 (Figure 4D). This led us to hypothesize that phosphorylated STAT3 may control the transcription of COL17 during wound repair. To confirm the effect of activated STAT3 on COL17 transcription and keratinocyte activation, we inhibited STAT3 activation using S3I-201, a small molecule that inhibits STAT3 phosphorylation (Figure 4E). Treatment with S3I-201 significantly reduced COL17 expression and impaired keratinocyte migration, proliferation, and adhesion (Figure 4, F–H). Moreover, the application of S3I-201 to normal murine wounds decreased COL17 expression and reduced epidermal proliferation, wound reepithelialization, and the wound closure rate (Figure 5). Similar results have been observed under the knockdown of STAT3 with siRNA (Supplemental Figure 6, A–D). Additionally, no additional effects except for cell adhesion were produced by STAT3 inhibition in COL17-knockdown cells, suggesting that COL17 is indeed a major downstream effector gene of STAT3 (Supplemental Figure 8). Collectively, these findings indicate that STAT3 played a pivotal role in regulating COL17 levels at the wound edge, thereby influencing keratinocyte activation during wound reepithelialization.

### COL17 expression is enhanced by PKM2 nuclear translocation through STAT3 activation in keratinocytes during wound healing.

**Figure 6:**
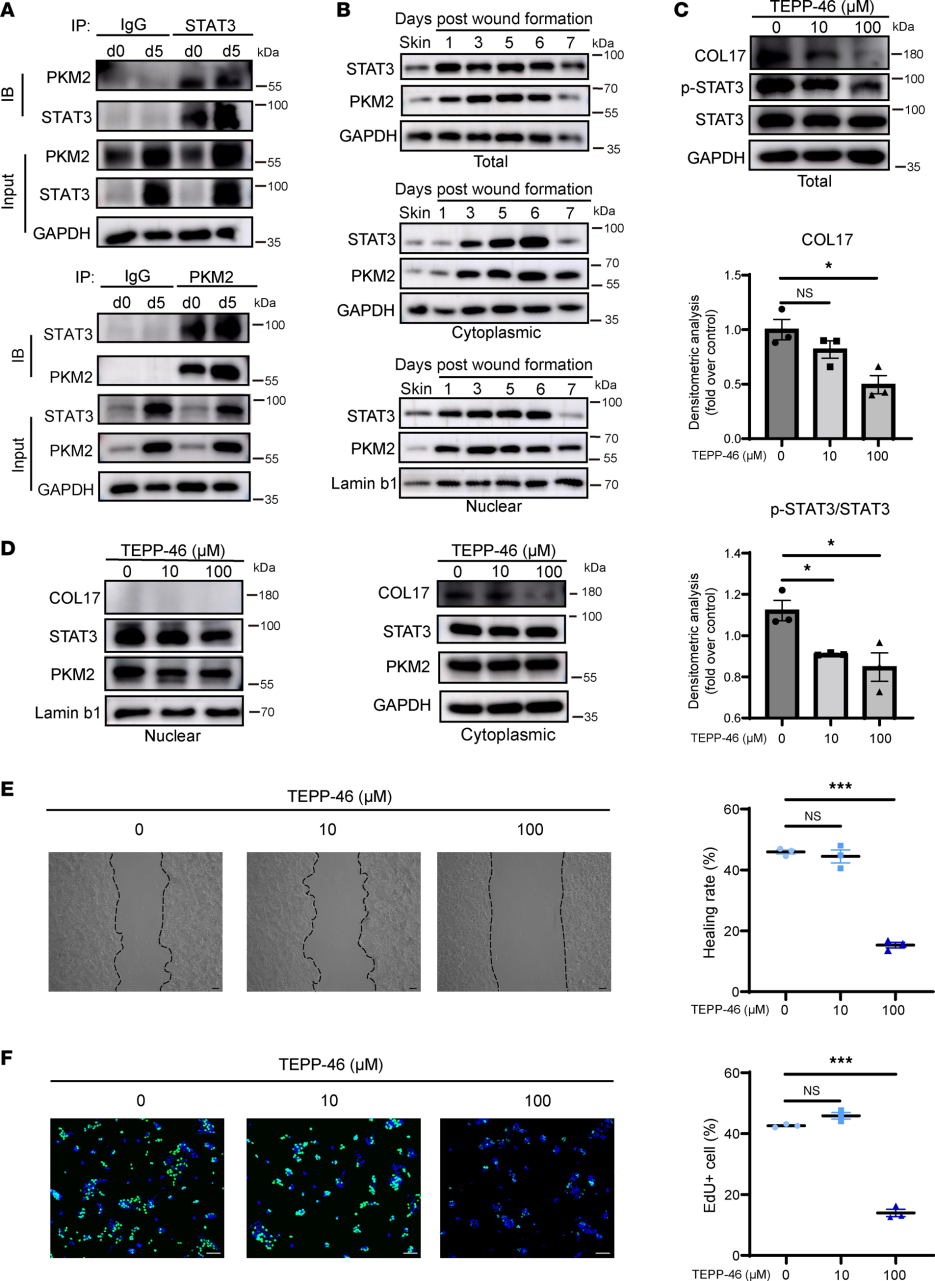
PKM2 promotes STAT3 phosphorylation and cotranslocation into the nucleus and activates keratinocytes. (**A**) CoIP analysis of the binding of PKM2 and STAT3 in murine wound edge tissues on PWD 0 and 5. (**B**) Chronological protein expression levels of total, cytoplasmic, and nuclear PKM2 and STAT3 in wound edge tissues from PWD 0 to 7. (**C**) Western blot and quantification analysis of COL17 and phosphorylated and total STAT3 in HaCaT cells treated with different concentrations of TEPP-46. (**D**) Protein expression levels of COL17, STAT3, and PKM2 in the nucleus or cytoplasm of HaCaT cells treated with different concentrations of TEPP-46. (**E**) Wound-healing assay and quantification analysis of HaCaT cells treated with different concentrations of TEPP-46 (scale bar: 100 μm). (**F**) EdU (green) proliferation assay and quantification analysis of HaCaT cells treated with different concentrations of TEPP-46 (scale bar: 100 μm). The data are presented as the mean ± SEM (*n* = 3 independent experiments). *P < 0.05, **P < 0.01, ***P < 0.001. ANOVA with Tukey’s test was used to compare multiple groups.

Although STAT3 has been identified as an upstream regulator of COL17, the mechanism underlying STAT3 activation during wound healing has remained unclear. Upon further analysis of the DNA pull-down high-throughput data, PKM2 emerged as a candidate of interest due to its role as a glycolytic enzyme with protein kinase activity capable of activating gene transcription by phosphorylating STAT3 (13). PKM2 binding to the COL17 promoter was confirmed (Supplemental Figure 2A), yet luciferase assays revealed a negative influence of PKM2 on COL17 transcription in the absence of STAT3 (Supplemental Figure 2B), suggesting that PKM2 may bind to the COL17 promoter through mediation by STAT3 to activate transcription. Subsequently, CoIP assays confirmed the binding of PKM2 to STAT3 (Figure 6A). Robust binding between PKM2 and STAT3 was observed compared with that of the IgG controls at the wound edges on PWD 5. Further analysis determined total, cytoplasmic, and nuclear PKM2 and STAT3 protein levels, which showed that both cytoplasmic and nuclear PKM2 and STAT3 increased during the early stages of wound healing. This observation further supported the notion that total PKM2 increased during wound healing, some of which may translocate with STAT3 to the nucleus to regulate the gene expression of COL17 (Figure 6B and Supplemental Figure 7A).

**Figure 7:**
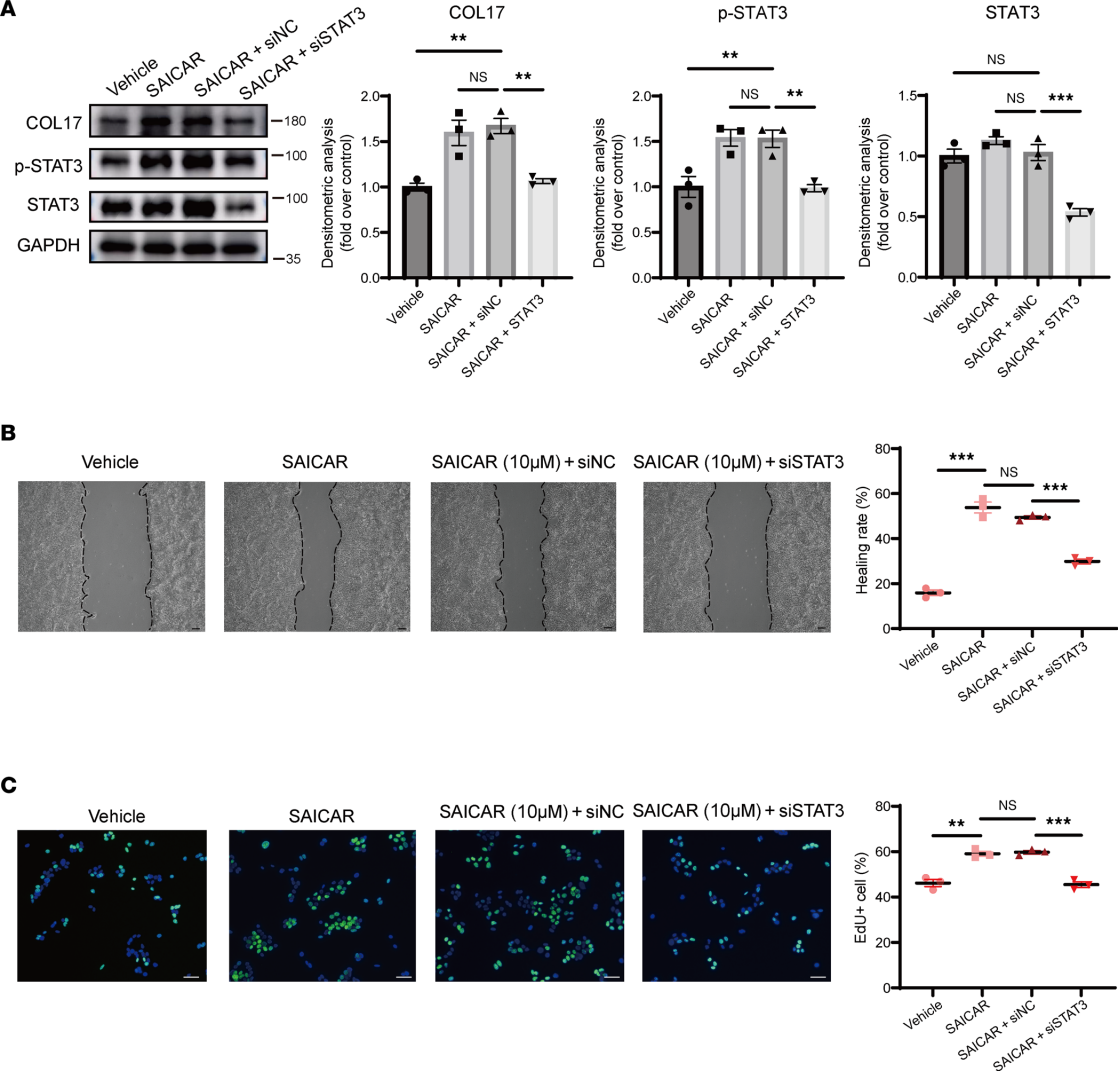
PKM2 promotes keratinocyte activation by STAT3 phosphorylation. (**A**) Western blot analysis of COL17 and phosphorylated and total STAT3 in HaCaT cells treated with vehicle, SAICAR, SAICAR plus siNC, or SAICAR plus siSTAT3. (**B**) Wound-healing assay and quantification analysis of HaCaT cells treated with vehicle, SAICAR, SAICAR plus siNC, or SAICAR plus siSTAT3 (scale bar: 100 μm). (**C**) EdU (green) proliferation assay and quantification analysis of HaCaT cells treated with vehicle, SAICAR, SAICAR plus siNC, or SAICAR plus siSTAT3 (scale bar: 100 μm). The data are presented as the mean ± SEM (*n* = 3 independent experiments). **P < 0.01, ***P < 0.001. ANOVA with Tukey’s test was used to compare multiple groups.

To examine the effect of PKM2 nuclear translocation on COL17 expression and keratinocyte behavior, TEPP-46 was used to inhibit PKM2 nuclear translocation. Treatment with 100 μM TEPP-46 significantly reduced the nuclear levels of PKM2 and STAT3, resulting in decreased COL17 expression (Figure 6, C and D, and Supplemental Figure 7B). The cell migration and proliferation of keratinocytes were also significantly inhibited (Figure 6, E and F). Conversely, the PKM2 nuclear translocator SAICAR promoted these phenotypes (Supplemental Figure 3, A–C). Interestingly, no significant difference in cell adhesion was observed between the TEPP-46-treated group and the control group, and a negative effect was observed in the SAICAR-treated groups (Supplemental Figure 4), which may be attributed to the multifunctionality of PKM2 in different cell signaling pathways. Overall, knockdown of total PKM2 in keratinocytes resulted in reduced phosphorylation of STAT3 and decreased COL17 expression, which in turn inhibited keratinocyte migration, proliferation, and adhesion (Supplemental Figure 6, E–H). To further elucidate whether the effect of PKM2 translocation on keratinocytes was mediated by STAT3, we investigated whether STAT3 inhibition could block the effect of SAICAR on the keratinocyte phenotype (Figure 7A and Supplemental Figure 3D). Treatment with SAICAR increased COL17 expression and stimulated keratinocyte migration and proliferation, and these effects were mitigated by STAT3 knockdown (Figure 7, B and C). In alignment with our in vitro findings, the in vivo study demonstrated that SAICAR significantly accelerated wound closure in mice. Conversely, TEPP-46 did not exhibit a notable inhibitory effect on wound healing (Supplemental Figure 5). The underlying mechanisms responsible for this confusing result warrant further investigation. Overall, these findings indicate that nuclear PKM2 increased during wound healing, promoting STAT3 phosphorylation and triggering its nuclear translocation, thereby promoting COL17 expression and activating keratinocytes.

### PKM2 nuclear translocator SAICAR induces COL17 expression to enhance impaired wound healing.

**Figure 8:**
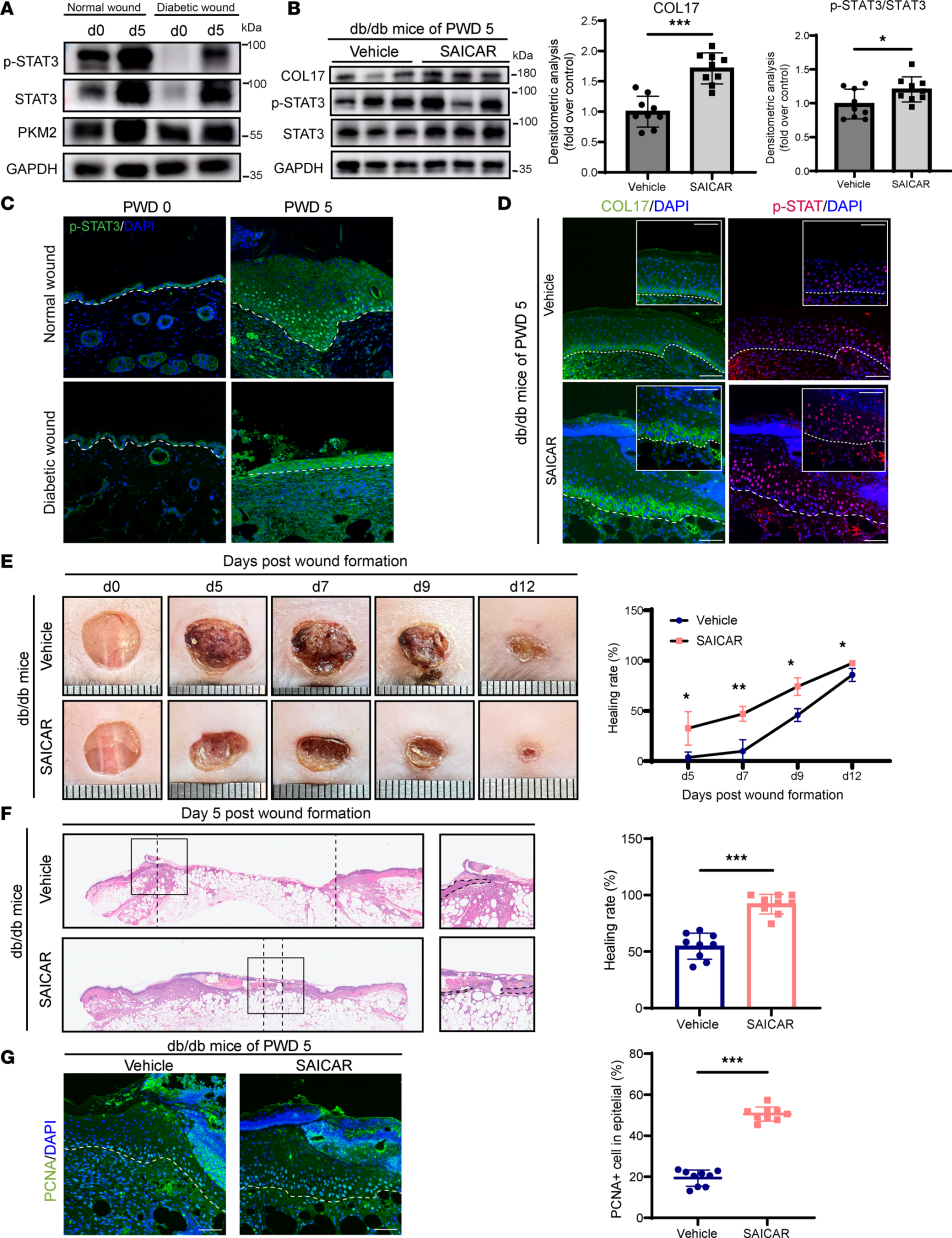
Topical injection of SAICAR ameliorates impaired wound healing in db/db mice. (**A**) Protein expression levels of PKM2 and phosphorylated and total STAT3 in normal and diabetic wound edge tissues on PWD 0 and 5. (**B**) Western blot and quantification analysis of COL17 and phosphorylated and total STAT3 in wound edges on PWD 5 in db/db mice treated with vehicle or SAICAR. (**C**) Representative immunofluorescence images of phosphorylated STAT3 in normal and diabetic wound edge tissues on PWD 0 and 5. (**D**) Representative immunofluorescence images of COL17 (green) and phosphorylated STAT3 (red) expression in wound edges on PWD 5 in db/db mice treated with vehicle or SAICAR. (**E**) Representative chronological images of wounds and analysis of the wound area healing rate in db/db mice treated with vehicle or SAICAR. (**F**) Representative H&E images of wounds and analysis of the wound gap healing rate on PWD 5 in db/db mice treated with vehicle or SAICAR. (**G**) Representative immunofluorescence images of PCNA (green) and the percentage of PCNA-positive cells at the wound edges on PWD 5 in db/db mice treated with vehicle or SAICAR. The data are presented as the mean ± SD (*n* = 9 independent animals). *P < 0.05, **P < 0.01, ***P < 0.001. Independent-sample *t* test was used for comparisons between the 2 groups and ANOVA with Tukey’s test was used to compare multiple groups.

Previous reports have indicated that STAT3 is inhibited in diabetic foot ulcers (14). This observation was corroborated in murine diabetic wounds, where both STAT3 expression and phosphorylation were suppressed on both PWD 0 and PWD 5 (Figure 8, A and C). Given the ability of the PKM2 nuclear translocator SAICAR to restore COL17 expression, thus rescuing impaired migration and proliferation in STAT3-deficient keratinocytes, we investigated whether SAICAR could alleviate the impaired wound-healing phenotype in diabetic mice. Initially, we observed a significant increase in phosphorylated STAT3 and COL17 expression in diabetic wounds treated with SAICAR (Figure 8, B and D). Consistent with our hypothesis, the topical application of SAICAR to both normal and diabetic wounds enhanced proliferation around the wound edge, resulting in accelerated reepithelialization and wound healing (Figure 8, E–G, and Supplemental Figure 5A). Together, these findings underscore the therapeutic potential of manipulating the PKM2/STAT3/COL17 pathway for improving the healing of chronic wounds.

## Discussion

Individuals experiencing impaired wound healing face a reduced quality of life and may even experience fatal outcomes. In this study, we elucidated the regulatory role of COL17 during cutaneous wound healing, proposed a potential pathway regulating COL17 expression, and identified a drug capable of inducing COL17 expression for treating chronic wounds. Upon injury, PKM2 interacted with the TF STAT3, leading to its translocation into the cell nucleus, where it bound to the promoter region of COL17. The resulting upregulation of COL17 promoted keratinocyte proliferation and migration, thereby accelerating wound reepithelialization. Chronic wounds often exhibit significant impairment of this pathway, but treatment with the PKM2 nuclear translocator SAICAR effectively enhanced wound repair in diabetic wounds.

Previous studies have reported an increase in COL17 expression following injury (5, 7). However, the specific expression pattern of COL17 in full-thickness excisional wounds has not been fully elucidated. Our data indicated that COL17 transcription continued to increase until PWD 7, while the protein level peaked on PWD 5 and subsequently returned to normal levels. Additionally, previous investigations have noted extensive COL17 expression throughout the entire wound epithelium on PWD 3, whereas it is restricted to the epithelial tongues on PWD 6 (5). These observations establish a spatiotemporal map of COL17 expression following the onset of wound healing, suggesting a triggering factor that initiates COL17 transcription for reepithelialization. However, the protein expression level of COL17 declines in the later stages of wound healing, which may be attributed to posttranscriptional regulation of mRNA, low translation efficiency, isoform variations, or even technical limitations. Further investigations utilizing spatial and single-cell transcriptomics at sequential phases of wound healing would provide additional insights into this spatiotemporal dynamic.

Compelling evidence suggests that COL17 is crucial for modulating epithelial cell behaviors. In terms of proliferation, COL17A1 serves as a reliable marker for epithelial stem cells, reflecting individual cellular potential and possession of the quality of self-renewal (6, 7). Regarding migration, the effect of COL17 on cell motility varies, affecting two specific aspects: velocity and orientation. Previous studies have shown that cell lines subjected to shCOL17 exhibit reduced migratory velocity (9). Moreover, COL17-KO primary epithelial cells display a dysregulated migration direction, accompanied by the formation of destabilized lamellipodia (8). Our current findings demonstrated that COL17 knockdown via siRNA alleviated the migration, proliferation, and adhesion of keratinocytes. Conversely, overexpression of COL17A1 had the opposite effect of enhancing these activities. A study using a mouse tail wound model revealed that hemidesmosome instability due to COL17 KO leads to defective wound healing, while forced expression of human COL17 by basal keratinocytes in mice promotes wound healing (6). In our study, we expanded upon the literature by overexpressing COL17 in db/db mice and showed that COL17 improved impaired wound healing by accelerating reepithelialization.

The intrinsic drivers of dynamic changes in COL17 expression during wound healing are poorly understood. Multiple studies have revealed the posttranscriptional regulation of COL17 in wound healing or hemidesmosome-defective diseases. TIMP1 hinders COL17 proteolysis by suppressing its metalloproteinase activity (15). COL17 distribution is influenced by Wnt signaling via COL17 phosphorylation (16). In an effort to gain further insight into the transcriptional regulation of COL17, we conducted a DNA pull-down assay combined with LC-MS/MS analysis. Our study revealed that the TF STAT3 and the glycolytic enzyme PKM2 exhibited differential binding to the COL17 promoter between wounded and unwounded skin. PKM2 is a crucial enzyme in glycolysis. The involvement of PKM2-mediated glycolysis has been well established in diseases related to regeneration and inflammation (17). PKM2 expression increases during the inflammatory phase of cutaneous wound healing in keratinocytes and macrophages (18). Macrophages that have increased pyruvate kinase activity undergo a transition toward a reparative phenotype and accelerate wound healing (19). In addition to its well-established role as a glycolytic enzyme in the cytoplasm, there have been ample hints that PKM2, after transitioning from a tetramer to a dimer, translocates into the nucleus and has protein kinase activity, which directly regulates gene transcription. In the nucleus, PKM2 interacts with phosphorylated β-catenin, enhancing the transactivation activity of β-catenin as a cofactor (20). PKM2 also functions as an epigenetic regulator to phosphorylate histone H3 at T11 (21). Previous studies have shown that the tightly orchestrated regulation of the TF STAT3 in epithelial, immune, and stromal cells is critical for wound healing and tissue repair (22). PKM2 phosphorylates STAT3 at Y705, inducing the nuclear translocation of STAT3 (13). Additionally, PKM2 can engage with nuclear STAT3, thus augmenting its transactivation (23). Therefore, PKM2-catalyzed STAT3 activation is a potential mechanism for regulating the expression of COL17. Here, our study established a pathway linking the above studies, elucidating a network between the upstream enzyme PKM2, the transcriptional regulator STAT3, and the downstream effector COL17 in the process of wound healing. During the wound-healing process, PKM2 promoted STAT3 phosphorylation and nuclear translocation, leading to increased expression of COL17. This, in turn, regulated keratinocyte behavior and promoted wound reepithelialization. Notably, EGFR activation has been shown to induce PKM2 translocation into the nucleus in human cancer cells (20). EGFR signaling is known to play a role in wound healing by accelerating wound reepithelialization (3). This finding suggested that EGF may serve as a potential trigger for PKM2 nuclear translocation in epithelial cells, further elucidating a comprehensive pathway for the immediate regulation of COL17 after injury. However, the regulatory network controlling COL17 expression likely extends beyond the pathway identified in our study. Investigations into other regulators, such as epigenetic modifications or translational regulations, may identify additional targets for intervention.

In terms of the clinical translation of these findings, pharmacological agents capable of inducing COL17 expression hold promise as therapies for chronic wounds. Two chemicals, Y27632 and apocynin, have been identified for their ability to induce COL17 expression, albeit with unclear regulatory mechanisms. Intriguingly, they have been shown to promote wound repair in a manner similar to the effect of COL17 overexpression in mice (6). In our present study, we identified SAICAR, a chemical compound known to stimulate PKM2 nuclear translocation (24, 25), as an inducer of COL17 expression through the PKM2-mediated mechanism described earlier. This discovery offers immediate medicinal advantages for accelerating wound repair. Curiously, TEPP-46, a PKM2 tetramer-promoting agent that traps PKM2 in the cytoplasm (26), did not significantly inhibit wound healing (Supplemental Figure 5B). In fact, it has been reported that the combined application of TEPP-46 and a Wnt signaling activator greatly enhances wound closure. These observed outcomes may be attributed to the modulation of cytoplasmic PKM2 by TEPP-46. In effector cells other than epithelial cells, such as macrophages, PKM2 plays a crucial role in cell function through its pyruvate kinase activity in the cytoplasm (19). Compared with TEPP-46, SAICAR has minimal effects on cytoplasmic PKM2, ensuring its effectiveness in wound healing.

In general, we elucidated the precise expression pattern and pivotal role of COL17 in wound healing. Notably, our study revealed a transcriptional regulatory mechanism involving COL17, facilitated by the interaction between PKM2 and STAT3. Specifically, PKM2 and STAT3 jointly translocate into the nucleus, leading to enhanced COL17 transcription. Furthermore, we identified SAICAR, a drug with COL17-inducing properties, which effectively ameliorates impaired wound healing. These findings provide valuable insights for advancing strategies aimed at enhancing skin repair.

## Methods

### Sex as a biological variable.

Our study examined male mice because male animals exhibited less variability in phenotype. It is unknown whether the findings are relevant for female mice.

### Animal ethics.

C57BL/6J mice aged 6 weeks and weighing 20–25 g were purchased from Shanghai Jihui Experimental Animal Breeding Co. C57BL/6J-db/db mice (a genetically obese mouse model that carries a mutation in the leptin receptor, leading to severe obesity, insulin resistance, and type 2 diabetes) aged 6 weeks and weighing 40–45 g were purchased from Cyagen Biosciences. All animals were maintained in the Animal Facility of Shanghai Ninth People’s Hospital. At the conclusion of the in vivo experiment, the animals were euthanized in accordance with the Canadian Council on Animal Care (CCAC) guidelines on: euthanasia of animals used in science.

### Excisional wound models.

The animals were euthanized with Zoletil-50 combined with dexmedetomidine (80/0.75 mg/kg). An 8 mm full-thickness excisional wound was developed through both skin and panniculus carnosus muscle on the central dorsal skin for observation (27). Each wound was photographed at different time points, and wound size was calculated by ImageJ (NIH) software. Healing rate (%) = (wound area on day 0 – wound area on the indicated time)/wound area on day 0. The animals were euthanized at the indicated time, and wound edges of 1 mm around the wound were snap-frozen or collected in 4% paraformaldehyde for further analyses. As for drug treatment, 1 mM S3I-201 (HY-15146, MCE, a small-molecule inhibitor of the STAT3), 1 m M TEPP-46 (HY-18657, MCE, a small-molecule inhibitor specifically designed to promote the PKM2 tetrameric form and localize in cytoplasm), or 100 μM SAICAR (HY-126585, MCE, a key intermediate in the de novo purine biosynthesis pathway, which selectively activates the PKM2 dimeric form and localizes in the nucleus) in 100 μL 0.9% saline was injected, and 0.9% saline with the same concentration of DMSO was injected for controls. Each substance was injected at sites located outside the wound area. Five injections of 20 μL each per wound were administered once a day the next day. The injections were carried out until the materials collection was completed. A flow chart and methods of the wound model can be referred to Supplemental Figure 1.

### RNA purification and RT-qPCR.

Total RNA was extracted using TRIzol reagent (Solarbio). Reverse transcreation was performed using the RT reagent kit (Takara). RT-qPCR was performed with QuantStudio 6 Flex (Thermo Fisher) using SYBR qPCR master mix (Vazyme) according to the manufacturer’s instructions. mRNA quantification was performed with the ΔΔCT method using GAPDH for normalization. The primers used in this study were as follows: GAPDH, forward, 5′-AGGTCGGTGTGAACGGATTTG-3′; reverse, 5′-TGTAGACCATGTAGTTGAGGTCA-3′; Mus musculus Col17a1, forward, 5’-AAGTCACCGAGAGAATTGTCAC-3′; reverse, 5′-AGAGAGCCTGTCTTAGCATATCC-3′.

### Western blot assay.

Tissues and cultured cells were lysed for 30 minutes with RIPA lysis buffer supplemented with phenyl methane sulfonyl fluoride (Solarbio) or with NE-PER Nuclear and Cytoplasmic Extraction Reagents (Thermo Fisher) according to the manufacturer’s instructions. 20 μg protein was electrophoresed and electroblotted to polyvinylidene difluoride membranes (Millipore). The membranes were blocked with 5% nonfat milk for 1 hour at room temperature. The separated membranes were then incubated with primary antibodies: anti-GAPDH (10494-1-AP, Proteintech, 1:10,000), anti-COL17 (A4808, ABclonal, 1:500), anti-STAT3 (10253-2-AP, Proteintech, 1:2,000), anti-pSTAT3 (no. 9145, CST, 1:2,000), anti-PKM2 (15822-1-AP, Proteintech, 1:2,000), and anti-Lamin B1 (12987-1-AP, Proteintech, 1:5,000) overnight at 4°C, followed by HRP-conjugated secondary antibody for 1 hour at room temperature on the next day. The membranes were last rinsed with ECL solution (NCM Biotech) and detected with Amersham Imager 600 (General Electric).

### H&E staining.

Tissues that were paraformaldehyde-fixed overnight and then paraffin-embedded were cut at a thickness of 5 mm. The paraffin sections were deparaffinized and rehydrated. The sections were then stained with hematoxylin solution (Harris) and eosin solution for analysis of wound histology with optical microscope (Nikon). Healing rate (%) = (wound gap on day 0 – wound gap on the indicated time)/wound gap on day 0.

### Immunofluorescence staining.

The paraffin sections were deparaffinized and rehydrated, and antigen retrieval was performed with Tris/EDTA buffer (pH 9.0). The sections were then permeabilized with 0.5% Triton X-100 for 15 minutes and blocked with 5% Donkey Serum Albumin (Solarbio) for 1 hour at room temperature. Samples were incubated with primary antibody against COL17 (ab184996, Abcam, 1:100), K14 (ab7800, Abcam, 1:1,000), PCNA (60097-1-Ig, Proteintech, 1:500), and pSTAT3 (no. 4113, CST, 1:200) overnight at 4°C, followed by Alexa Fluor 488 Goat anti-Rabbit and Alexa Fluor 555 Goat anti-Mouse secondary antibody (Invitrogen, 1:500) for 1 hour at room temperature. DAPI was used as a nuclear counter stain. Fluorescence was analyzed with an optical microscope (Nikon).

### Cells.

Immortalized human keratinocyte cell line (HaCaT, an immortalized human keratinocyte cell line derived from adult skin) and human embryonic kidney 293T cell line (HEK293T) were purchased from the National Collection of Authenticated Cell Cultures of China and cultured in DMEM (GIBCO) supplemented with 10% fetal bovine serum (GIBCO). The cells were grown in a humidified incubator with 5% CO_2_ at 37°C. HaCaT cells were seeded in 6- or 96-well plates and incubated overnight for further analysis. After starvation for 12 hours, cells were treated with different concentrations of chemicals S3I-201, TEPP-46, and SAICAR for 24 hours. HEK293T cells were used for lentivirus packaging.

### In vitro wound healing.

Cells were seeded into 6-well dishes and reached an 80%–90% confluence as a monolayer. Then, the cells were starved in serum-free basal medium for 22 hours, followed by treatment with mitomycin C (10 μg/mL) for 2 hours to arrest their proliferation. The wound was generated using a 200 μL pipette tip across the cell monolayer. After wounding, cells were treated with different concentrations of chemicals. Migration of cells into the wound was observed at 0 hours and 24 hours by Axio Vert.A1 microscope (Zeiss). Migration rate (%) = (wound gap at 0 hours – wound gap at 24 hours)/wound gap at 0 hours.

### EdU cell proliferation assay.

Cell proliferation was detected by the BeyoClick EdU Cell Proliferation Kit with the Alexa Fluor 488 kit (C0071S, Beyotime) according to the manufacturer’s instructions. The proportion of cells that incorporated EdU or Hoechst 33342 was determined by an Axio Vert.A1 microscope (Zeiss).

### Cell adhesion assay.

The 96-well plates were precoated with diluted Matrigel (354234, Corning, 1:10) for 1 hour at 37°C. Cells were then seeded on the coated plate at 2 × 10^5^ cells/mL and incubated for 2 hours for adhesion. For each group, half of the wells were washed with PBS to dismiss the nonadherent cells while the others remained unwashed. Cells were fixed, permeabilized, and stained with 0.1% crystal violet (Solarbio) for 30 minutes at room temperature. Stained cells were observed under a SMZ25 stereomicroscope (Nikon) and resolved in 1% SDS solution to measure the absorbance at 590 nm on a spectrophotometer (BioTek). Adhesion rate (%) = absorbance of adherent cells/absorbance of total cells.

### siRNA and plasmids transfection.

All transfections were performed using Lipofectamine 3000 reagent (no. L3000150, Thermo Fisher Scientific) according to the manufacturer’s protocol.

### Lentivirus packaging and transfection.

pGMLV-CMV-H_COL17A1(NM_000494.4) and pGMLV-CMV-m_COL17A1(NM_007732.3) plasmids were generated and lentiviral packaging was performed with the lentiviral packaging kit (Genomeditech) according to the manufacturer’s protocol. In vitro, HaCaT cells were plated in 6-well plates. After reaching 70% confluence, a medium containing lentivirus and polybrene (6 μg/mL) was added to the cells. After incubation for 24 hours, supernatants in the wells were replaced by DMEM for 24 and 48 hours for subsequent analyses. In vivo lentiviral injection was performed 1 month before incision. The area for the excisional wound was first marked at the center of the dorsal skin. The lentivirus for in vivo experiments was concentrated to a titer of 1.25 × 10^8–9^ TU/mL, measured by the Rapid Lentivirus Titration Cassette (BF06202, Biodragon). 100 μL concentrated virus was then intradermally injected with a 26-gauge needle and 100 μL syringe (Gaoge) at 5 points around the marked area. The successful overexpression of COL17 in the epithelial cells was later validated through Western blot and immunofluorescence analysis (Figure 3, D and E, and Supplemental Figure 9).

### DNA pull-down plus LC-MS/MS.

Nuclear proteins from wound edge tissues were extracted for DNA pull-down assays. Single-strand probes from the COL17 promoter for DNA pull-down were synthesized and labeled with biotin tags. Nuclear samples were then pulled down with Dynabeads C1 Streptavidin (Thermo) and probe complex overnight at 4°C and eluted. The captured proteins were then digested with trypsin and desalted for further LC-MS/MS analysis. Identification of the peptides binding to the probe was achieved using LC-MS/MS. LC-MS/MS raw data were searched and quantified using MaxQuant (version 2.0.1.0). Data from PWD 5 were compared with those from PWD 0; these proteins had a FCa = log_2_(FC), where FC is the ratio of the standardized spectral counts of the protein in PWD 5 to that in PWD 0. Proteins with an FCa > log_2_(1.5) and unique peptide ≥2 were considered of higher enrichment in PWD 5.

### Coimmunoprecipitation assay.

Cell lysates were collected using an ice-cold IP lysis buffer. The coimmunoprecipitation analyses were performed using Protein A/G Magnetic Beads (HY-K0202, MCE) according to the manufacturer’s protocol. Briefly, magnetic beads were prepared with 2 washes with binding/wash buffer. Anti-STAT3 antibody (60199-1-Ig, Proteintech), anti-PKM2 antibody (60268-1-Ig, Proteintech), and normal mouse IgG as negative control were diluted to the final concentration of 10 μg/mL with binding/wash buffer and incubated with the Protein A/G Magnetic Beads for 2 hours at 4°C. Samples containing the antigen were incubated with the Protein A/G Magnetic Beads-Ab complex for 2 hours at 4°C. Proteins were eluted with SDS-loading buffer, and magnetic beads were separated. Western blotting was performed as described above.

### ChIP assay.

ChIP assay was performed using the SimpleChIP Plus Enzymatic Chromatin IP Kit (no. 9005, CST) according to the manufacturer’s protocol. Briefly, tissues were cross-linked with formalin and disaggregated. Nuclei were prepared and chromatin was digested with micrococcal nuclease to a DNA length of approximately 150–900 bp. Immunoprecipitation was carried out overnight with anti-STAT3 antibody (no. 4904, CST), anti-PKM2 antibody (15822-1-AP, Proteintech), anti-H3 antibody as positive control, or normal rabbit IgG as negative control, followed by precipitation with magnetic beads. Five percent of the sample from each immunoprecipitation was reserved for input control. Chromatins were eluted from antibody/magnetic beads, DNA-protein crosslinks were reversed, and DNA from each sample was purified. Percentage input was measured by qRT-PCR using a titration of pooled input samples as a standard curve.

### Luciferase reporter assay.

The luciferase reporter containing the COL17 promoter (–1,920 kb to +80 kb) was conducted, and all PCR products were subcloned into pGL3-Basic (Promega). pGMLV-CMV-H_STAT3 and pGMLV-CMV-H_PKM plasmids were generated. Briefly, for the reporter assay, cells were plated in 24-well plates 1 day before transfection. The above overexpression, reporter, and pRL-TK plasmids were transfected according to the manufacturer’s instructions on individual experiments. Then, the cells were lysed and luciferase reporter activity was measured using the Luciferase Reporter system (Genomeditech) with Firefly luciferase values normalized to Renilla luciferase values.

### Statistics.

Data were analyzed with GraphPad Prism software, version 9.3.1. Each experiment was performed 3 times, and each in vivo experiment contained 9 independent animals. An independent-sample, 2-tailed *t* test was used for comparisons between the 2 groups. Levene’s test was performed to test the equality of variances. One-way ANOVA followed by a post hoc test (Tukey’s test) was used to compare multiple groups. Data are expressed as the mean ± SEM or SD. All *P* values of less than 0.05 were considered statistically significant.

### Study approval.

The animal use protocol has been reviewed and approved by the Laboratory Animal Ethics Committee in Ninth People’s Hospital Affiliated to Shanghai Jiao Tong University School of Medicine, and all procedures were performed in accordance with the *Guide for the Care and Use of Laboratory Animals* (National Academies Press, 2011).

### Data availability.

The datasets analyzed in this study are available from the public databases. Individual values for all other data are available in the Supporting Data Values file.

## Author contributions

Conception and design were provided by YG, YZ, Yangdan Liu, and QL. Study materials were provided or patients were recruited by YG, Yangdan Liu, YZ, CH, DSW, and QL. Collection and assembly of data were performed by Yangdan Liu, YG, CH, YZ, DW, JS, and Yuxin Liu. Data analysis and interpretation were performed by YG, YZ, and QL. Manuscript writing was performed by Yangdan Liu, YG, CH, YZ, and QL. All authors provided final approval of the manuscript.

## Supplementary Material

Supplemental data

Unedited blot and gel images

Supporting data values

## Version 1 01/22/2025

In-Press Preview

## Version 2 02/24/2025

Electronic publication

## Version 3 02/25/2025

The authorship contribution note was updated

## References

[1] Sen CK (2021). Human wound and its burden: updated 2020 compendium of estimates. Adv Wound Care (New Rochelle).

[2] Eming SA (2014). Wound repair and regeneration: mechanisms, signaling, and translation. Sci Transl Med.

[3] Rousselle P (2019). Re-epithelialization of adult skin wounds: Cellular mechanisms and therapeutic strategies. Adv Drug Deliv Rev.

[4] Natsuga K (2019). Life before and beyond blistering: The role of collagen XVII in epidermal physiology. Exp Dermatol.

[5] Jackow J (2016). Generation of a functional non-shedding collagen XVII mouse model: relevance of collagen XVII shedding in wound healing. J Invest Dermatol.

[6] Liu N (2019). Stem cell competition orchestrates skin homeostasis and ageing. Nature.

[7] Haensel D (2020). Defining epidermal basal cell states during skin homeostasis and wound healing using single-cell transcriptomics. Cell Rep.

[8] Hamill KJ (2011). Type XVII collagen regulates lamellipod stability, cell motility, and signaling to Rac1 by targeting bullous pemphigoid antigen 1e to alpha6beta4 integrin. J Biol Chem.

[9] Qiao H (2009). Collagen XVII participates in keratinocyte adhesion to collagen IV, and in p38MAPK-dependent migration and cell signaling. J Invest Dermatol.

[10] Fisher G, Rittié L (2018). Restoration of the basement membrane after wounding: a hallmark of young human skin altered with aging. J Cell Commun Signal.

[11] Jacinto A (2001). Mechanisms of epithelial fusion and repair. Nat Cell Biol.

[12] Yu H (2014). Revisiting STAT3 signalling in cancer: new and unexpected biological functions. Nat Rev Cancer.

[13] Gao X (2012). Pyruvate kinase M2 regulates gene transcription by acting as a protein kinase. Mol Cell.

[14] Sawaya AP (2020). Deregulated immune cell recruitment orchestrated by FOXM1 impairs human diabetic wound healing. Nat Commun.

[15] Nanba D (2021). EGFR-mediated epidermal stem cell motility drives skin regeneration through COL17A1 proteolysis. J Cell Biol.

[16] Hiroyasu S, Tsuruta D (2021). Stabilization of hemidesmosomal proteins: a possible key contributor to Wnt/β-Catenin pathway action in the skin. J Invest Dermatol.

[17] Yang W, Lu Z (2015). Pyruvate kinase M2 at a glance. J Cell Sci.

[18] Sych K (2023). Expression of PKM2 in wound keratinocytes is coupled to angiogenesis during skin repair in vivo and in HaCaT keratinocytes in vitro. J Mol Med (Berl).

[19] Wang J (2022). Lactylation of PKM2 suppresses inflammatory metabolic adaptation in pro-inflammatory macrophages. Int J Biol Sci.

[20] Yang W (2011). Nuclear PKM2 regulates β-catenin transactivation upon EGFR activation. Nature.

[21] Yang W (2012). PKM2 phosphorylates histone H3 and promotes gene transcription and tumorigenesis. Cell.

[22] Huynh J (2019). Therapeutically exploiting STAT3 activity in cancer - using tissue repair as a road map. Nat Rev Cancer.

[23] Damasceno LEA (2020). PKM2 promotes Th17 cell differentiation and autoimmune inflammation by fine-tuning STAT3 activation. J Exp Med.

[24] Keller KE (2012). SAICAR stimulates pyruvate kinase isoform M2 and promotes cancer cell survival in glucose-limited conditions. Science.

[25] Keller KE (2014). SAICAR induces protein kinase activity of PKM2 that is necessary for sustained proliferative signaling of cancer cells. Mol Cell.

[26] Anastasiou D (2012). Pyruvate kinase M2 activators promote tetramer formation and suppress tumorigenesis. Nat Chem Biol.

[27] Wang X (2013). The mouse excisional wound splinting model, including applications for stem cell transplantation. Nat Protoc.

