# Supplementary material for: PKM2-mediated collagen XVII expression is critical for wound repair
Source: JCI Insight. 2025 Jan 21;10(4):e184457. doi: 10.1172/jci.insight.184457 (PMC11856949; doi:10.1172/jci.insight.184457)
Supplement: Supplemental data [file jciinsight-10-184457-s285.pdf]

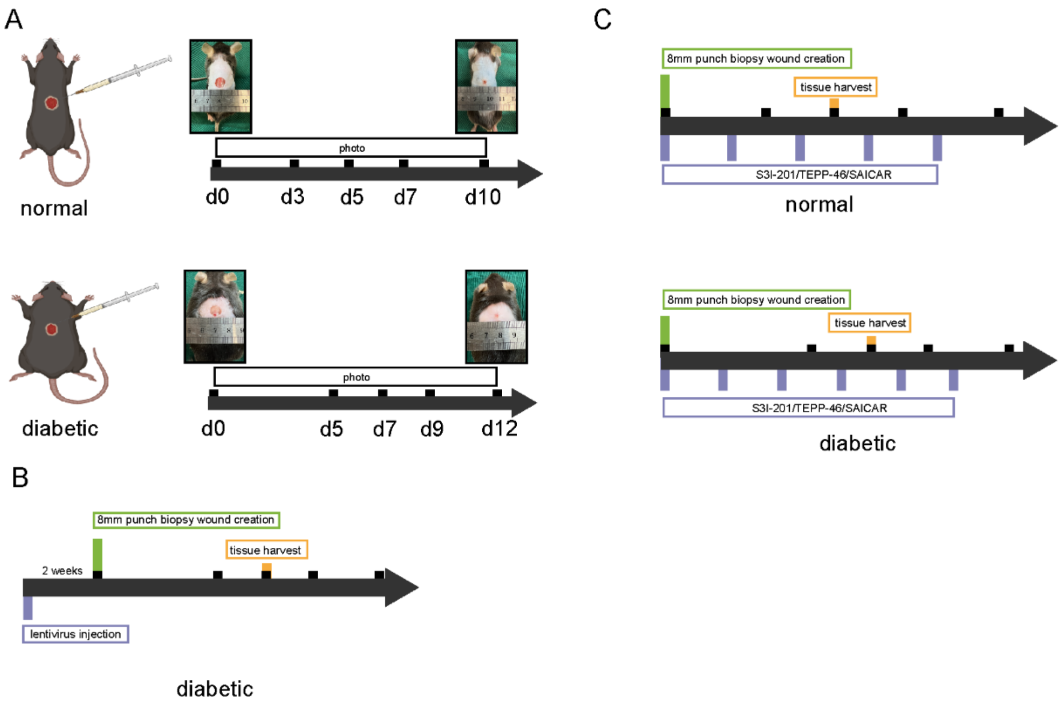

Figure S1 Strategy used to establish the murine models. (A) Schematic representation of the murine macrophotograph harvesting strategy for normal and diabetic mice. (B) Schematic representation of the lentivirus injection strategy used in diabetic mice. (C) Schematic representation of the S3I-201, TEPP-46 and SAICAR administration strategies in normal and diabetic mice.

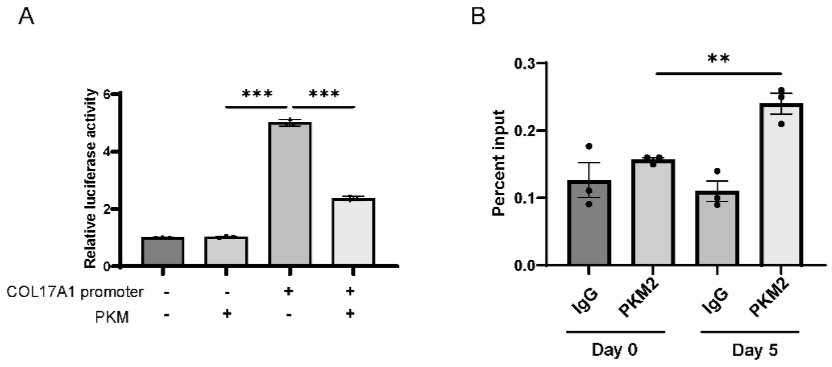

Figure S2 PKM2 does not directly promote COL17 transcription. (A) Luciferase reporter assay of COL17 transcriptional activity with or without PKM2 transfection. (B) ChIP analysis of PKM2 enrichment at the Col17a1 promoter in murine wound edge tissues on PWD 0 and 5. The data are presented as the means ± SEMs (n = 3 independent experiments). **P < 0.01, ***P < 0.001, ns= not significant. SEM: standard error of the mean.

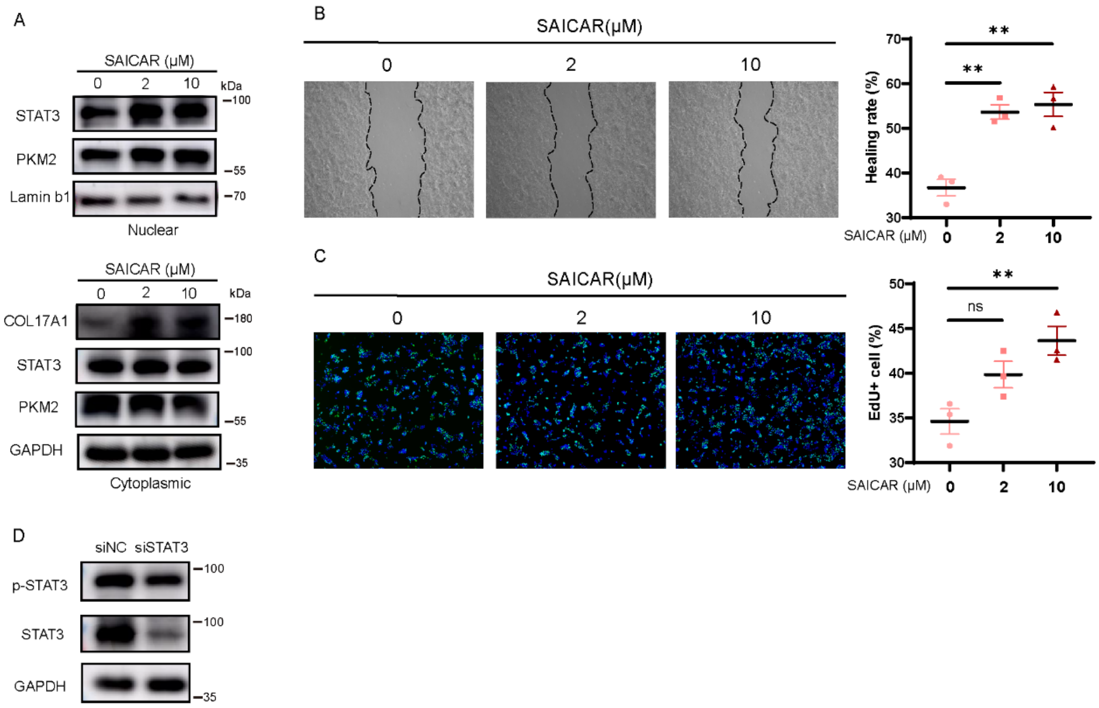

Figure S3 SAICAR promotes keratinocyte activation. (A) Western blot analysis of COL17, PKM2 and STAT3 in the nucleus or cytoplasm of HaCaT cells treated with different concentrations of SAICAR. (B) Wound healing assay and quantification analysis of HaCaT cells treated with different concentrations of SAICAR (scale bar=100 μm). (C) EdU (green) proliferation assay and quantification analysis of HaCaT cells treated with different concentrations of SAICAR (scale bar=100 μm). (D) Western blot analysis of COL17 and phosphorylated and total STAT3 in HaCaT cells treated with siSTAT3.The data are presented as the means ± SEMs (n = 3 independent experiments). **P < 0.01, ns= not significant. SEM: standard error of the mean.

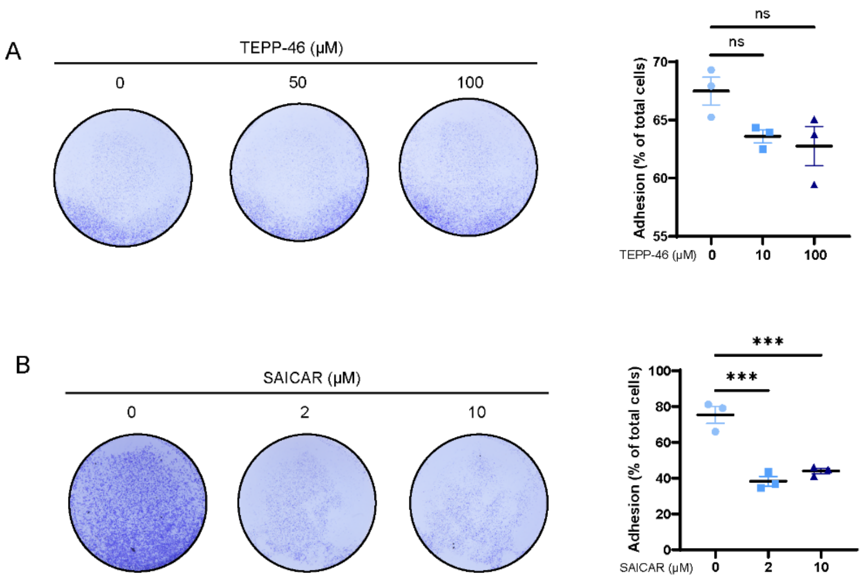

Figure S4 Effect of TEPP-46 or SAICAR on keratinocyte adhesion. (A) Adhesion assay and quantification analysis of HaCaT cells treated with different concentrations of TEPP-46. (B) Adhesion assay and quantification analysis of HaCaT cells treated with different concentrations of SAICAR. The data are presented as the means ± SEMs (n = 3 independent experiments). ***P < 0.001, ns= not significant. SEM: standard error of the mean.

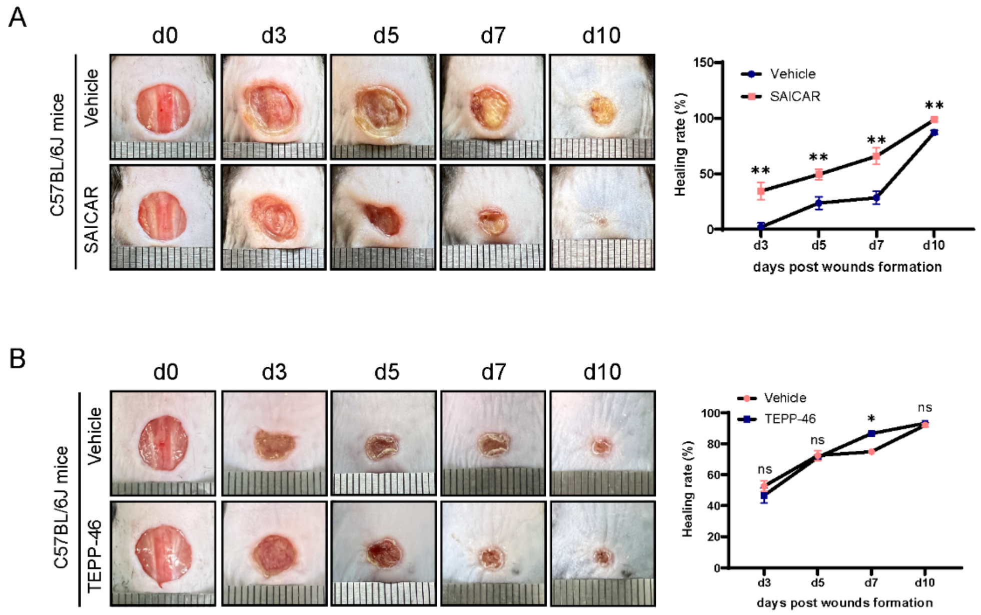

Figure S5 Effect of SAICAR or TEPP-46 on normal wound healing. (A) Representative chronological images of wounds and analysis of the wound area healing rate in C57BL/6J mice treated with vehicle or SAICAR. (B) Representative chronological images of wounds and analysis of the wound area healing rate in C57BL/6J mice treated with vehicle or TEPP-46. The data are presented as the means ± SDs (n = 3 independent animals). *P < 0.05, **P < 0.01, ns= not significant. SD: standard deviation.

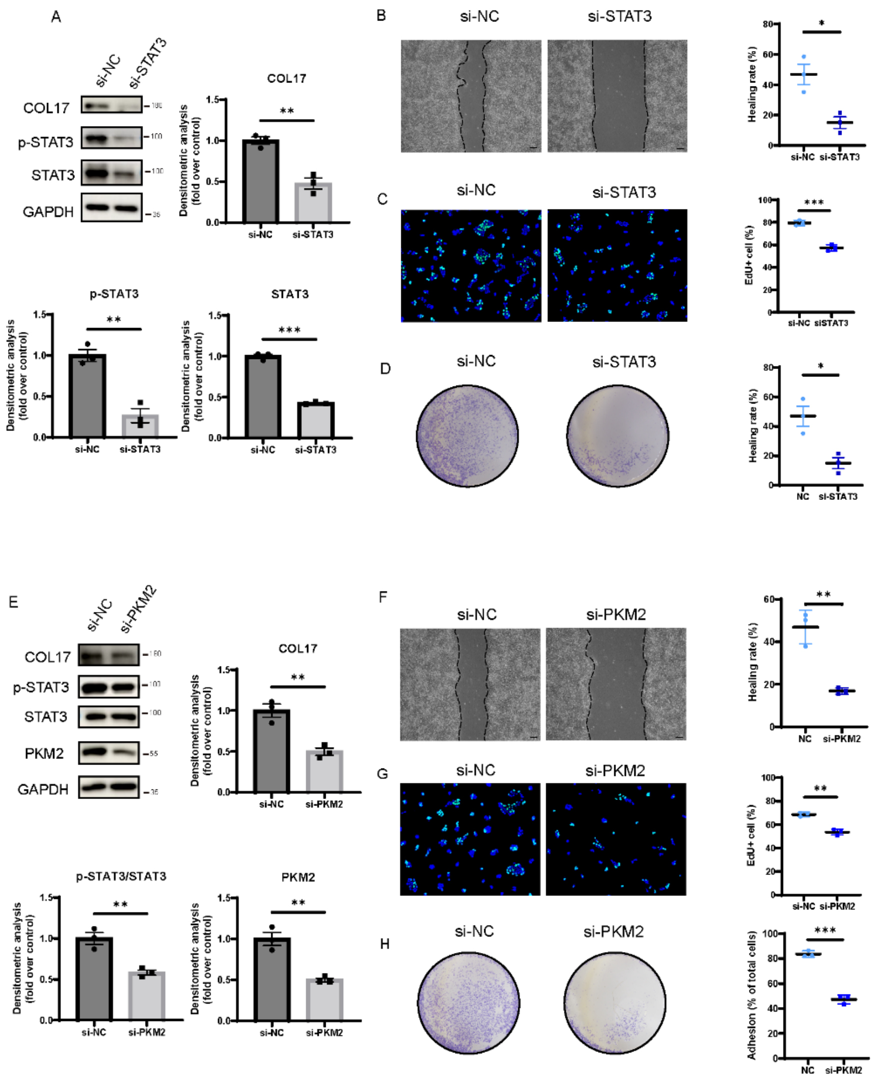

Figure S6 Effect of STAT3 KD and PKM2 KD on keratinocytes activation. (A) Protein expression level and quantification analysis of COL17, pSTAT3, STAT3 and PKM2 in HaCaT cells transfected with si-STAT3 or si-PKM2. (B) Relative mRNA expression level of COL17A1 in HaCaT cells transfected with si-STAT3 or si-PKM2. (C) Wound healing assay and quantification analysis of HaCaT cells with STAT3 or PKM2 KD (scale bar=100 μm). (D) EdU (green) proliferation assay and quantification analysis of HaCaT cells with STAT3 or PKM2 KD (scale bar=100 μm). (E) Adhesion assay and quantification analysis of HaCaT cells with STAT3 or PKM2 KD. The data are presented as the means ± SEMs (n = 3 independent experiments). *P < 0.05, **P < 0.01, ***P < 0.001. SEM: standard error of the mean.

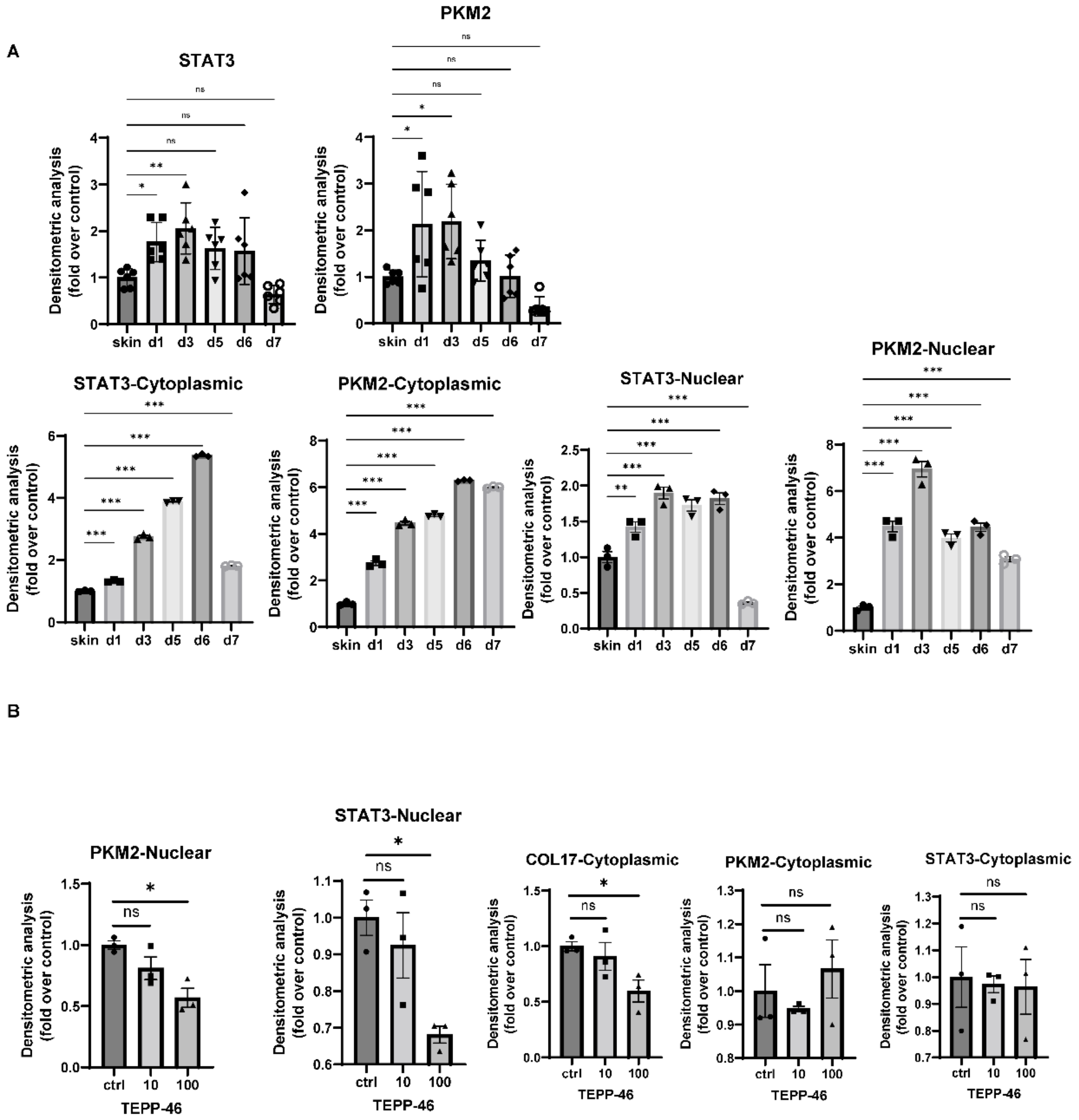

Figure S7 Quantification analysis of protein levels in Figure6

(A) Quantification analysis of protein expression levels of total, cytoplasmic and nuclear PKM2 and STAT3 in wound edge tissues from PWD 0 to 7. (B) Quantification analysis of Protein expression levels of COL17, STAT3 and PKM2 in the nucleus or cytoplasm of HaCaT cells treated with different concentrations of TEPP-46. The data are presented as the means ± SEMs (n = 3 independent experiments or 6 animals). *P < 0.05, **P < 0.01, ***P < 0.001, ns= not significant. SEM: standard error of the mean.

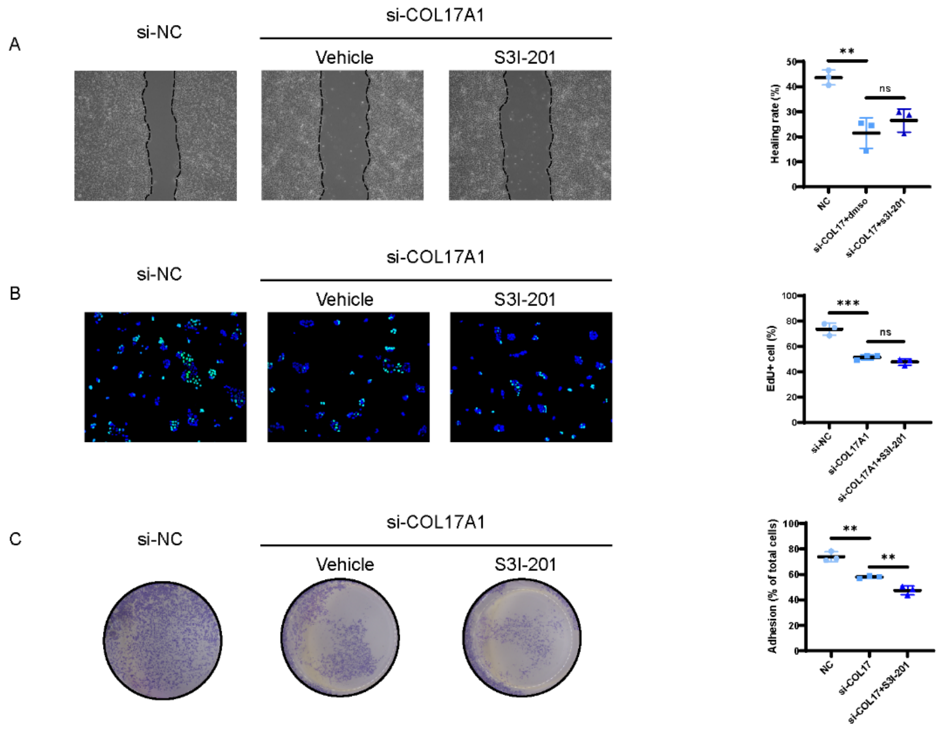

Figure S8 Effects of S3I-201 in COL17 KD cells on keratinocytes activation

(A) Wound healing assay and quantification analysis of HaCaT cells treated with siNC, siCOL17 plus vehicle or siCOL17 plus S3I-201 (scale bar= 100 μm). (B) EdU (green) proliferation assay and quantification analysis of HaCaT cells treated with siNC, siCOL17 plus vehicle or siCOL17 plus S3I-201 (scale bar= 100 μm). (C) Adhesion assay and quantification analysis of HaCaT cells treated with siNC, siCOL17 plus vehicle or siCOL17 plus S3I-201. The data are presented as the means ± SEMs (n = 3 independent experiments). **P < 0.01, ***P < 0.001, ns= not significant. SEM: standard error of the mean.

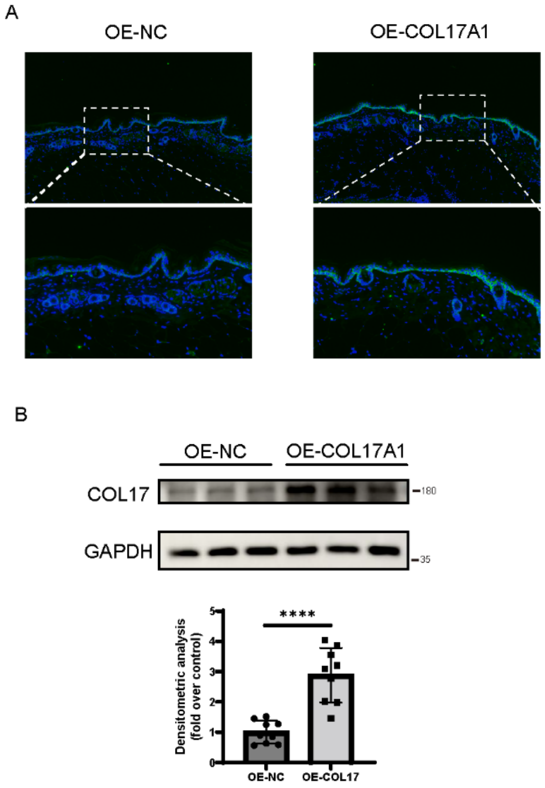

Figure S9 Verification of COL17 overexpression in mice

(A) Representative immunofluorescence images of COL17 (green) in the wounds of negative control (NC) or Col17a1 lentivirus-transfected mice at before incision (scale bar=20 μm). (B) Western blot and quantification analysis of COL17 in skin of mice treated with NC or Col17a1 lentivirus. The data are presented as the means ± SDs (n = 9 independent animals). ***P < 0.001, SD: standard deviation.

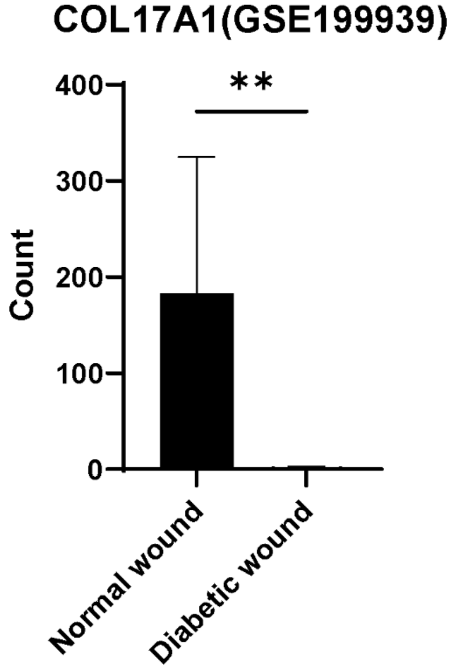

Figure S10 COL17A1 level in normal and diabetic wound of human tissues

Analysis of Col17a1 mRNA expression (data from GSE199939) in normal and diabetic wound of human tissues. The data are presented as the means ± SDs. **P < 0.01, SD: standard deviation.
