# Supplementary material for: PKM2-mediated collagen XVII expression is critical for wound repair
Source: JCI Insight. 2025 Jan 21;10(4):e184457. doi: 10.1172/jci.insight.184457 (PMC11856949; doi:10.1172/jci.insight.184457)

Fig 1D

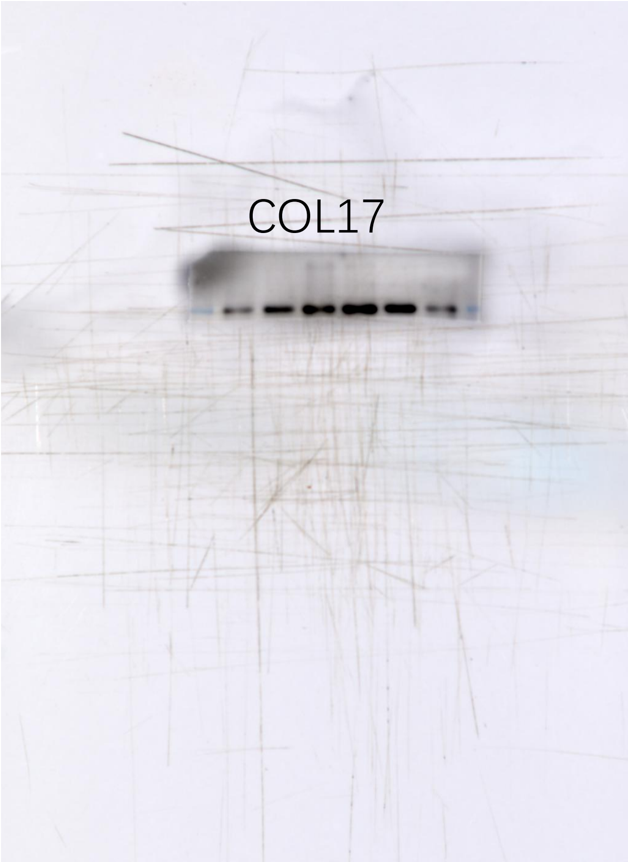

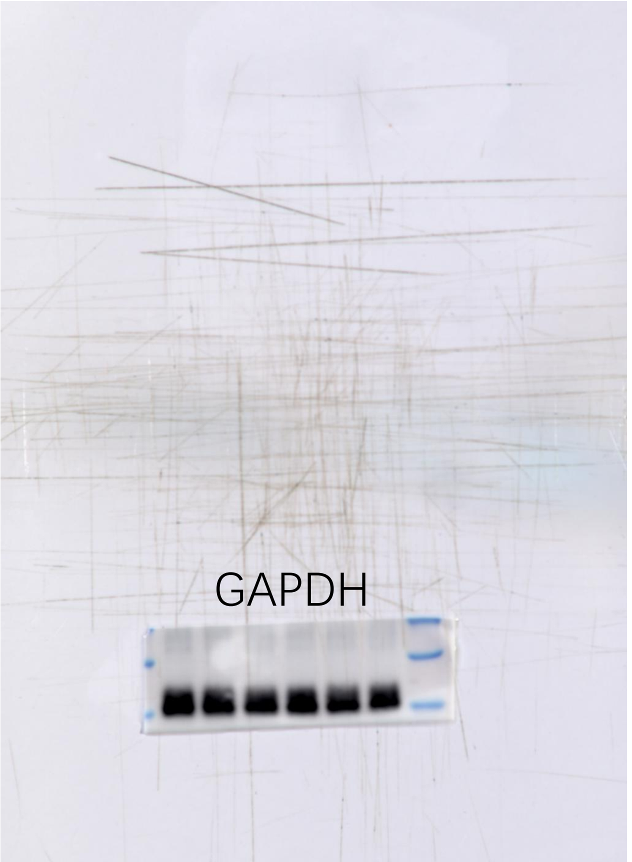

Fig 2A

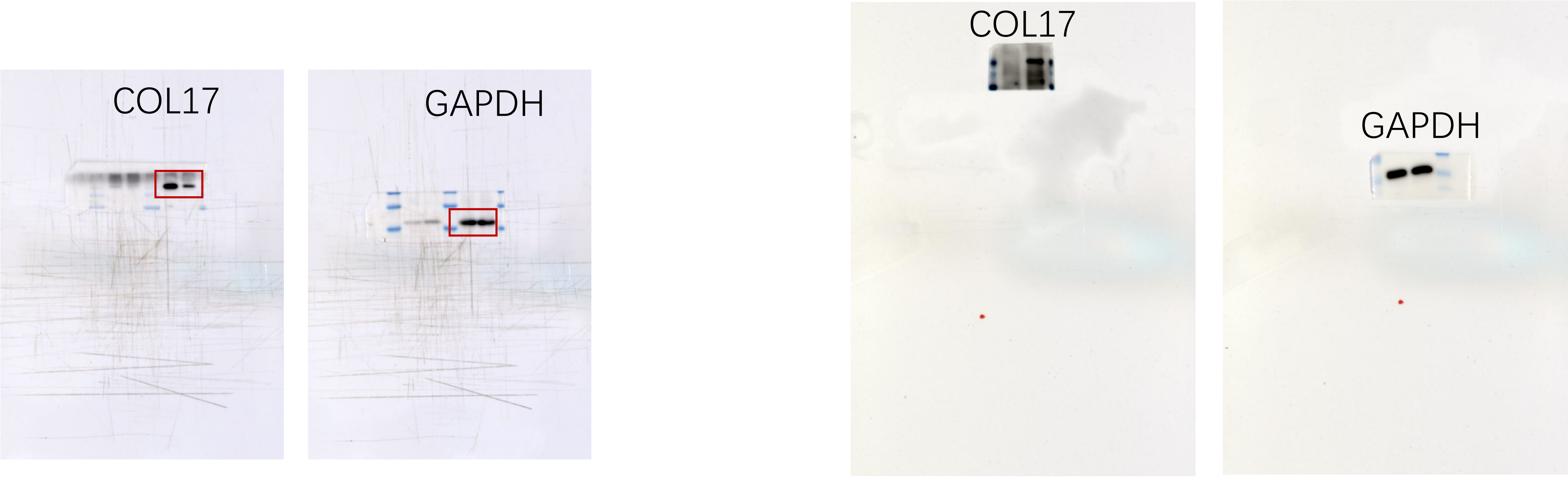

Fig 3C

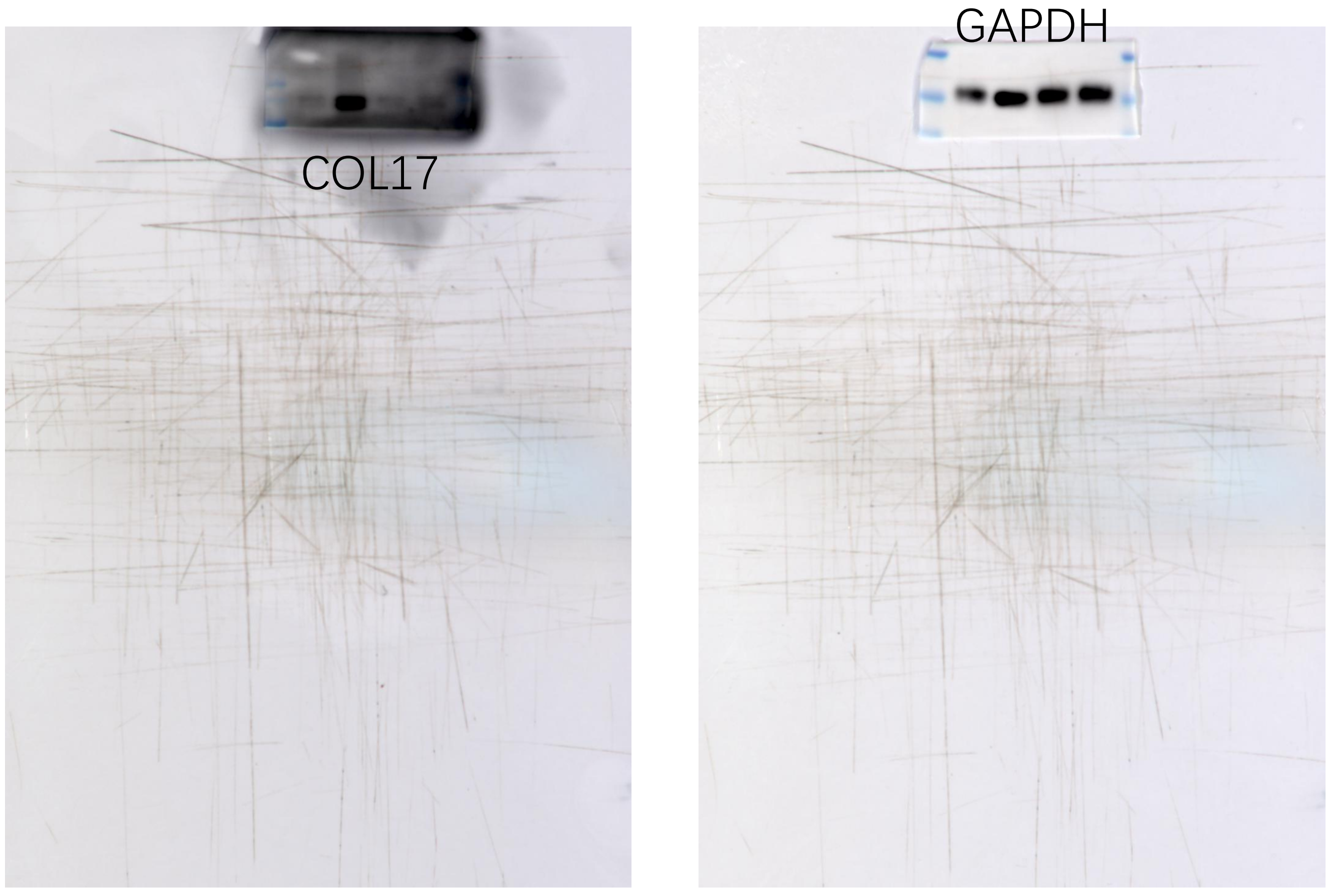

Fig 3E

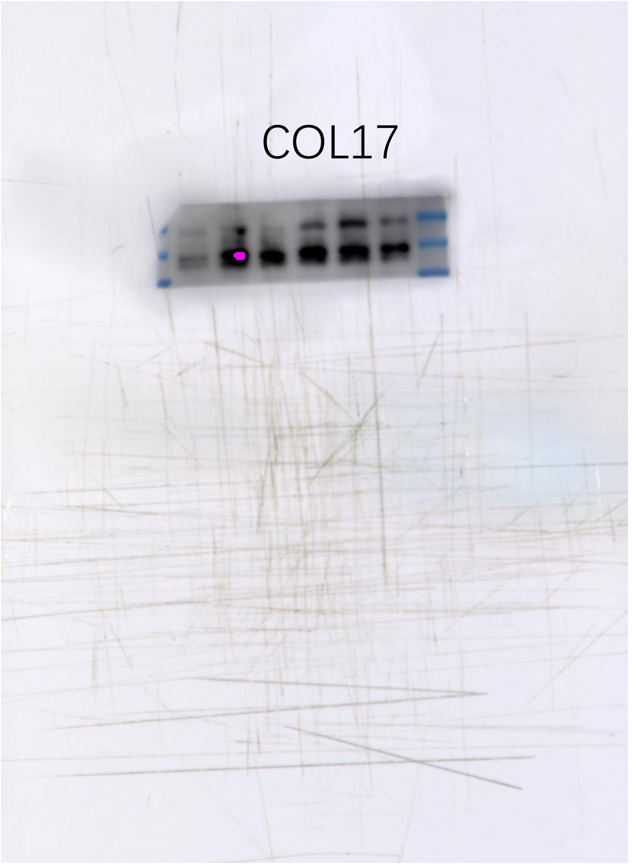

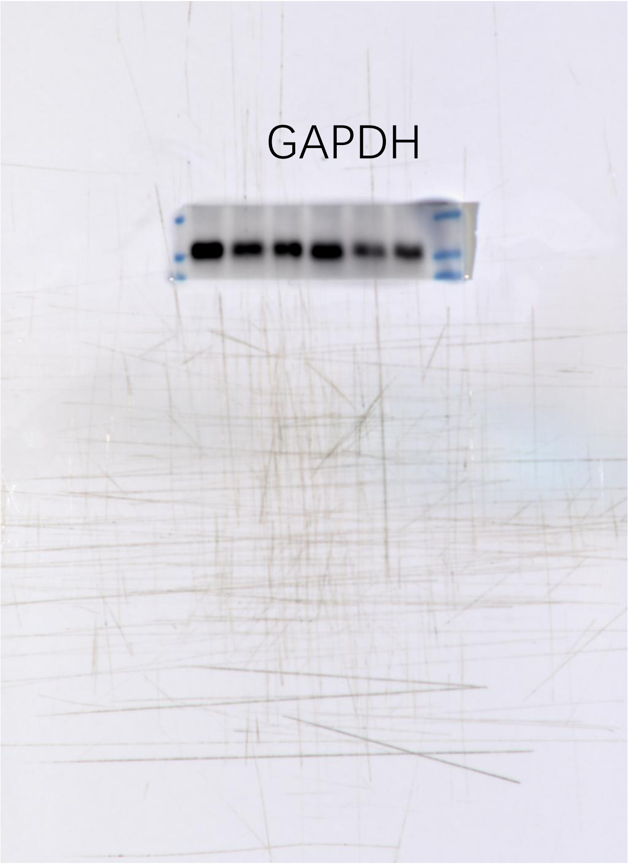

Fig 4D

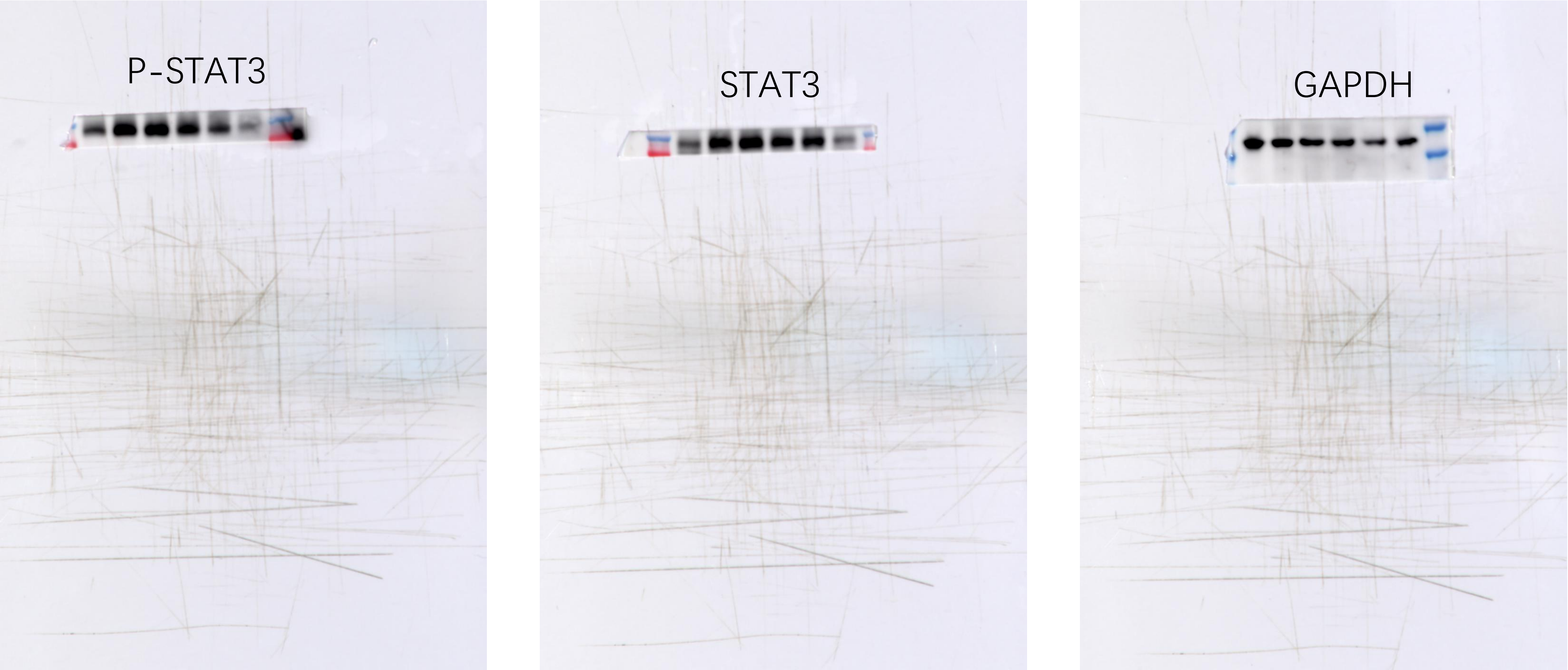

Fig 4E

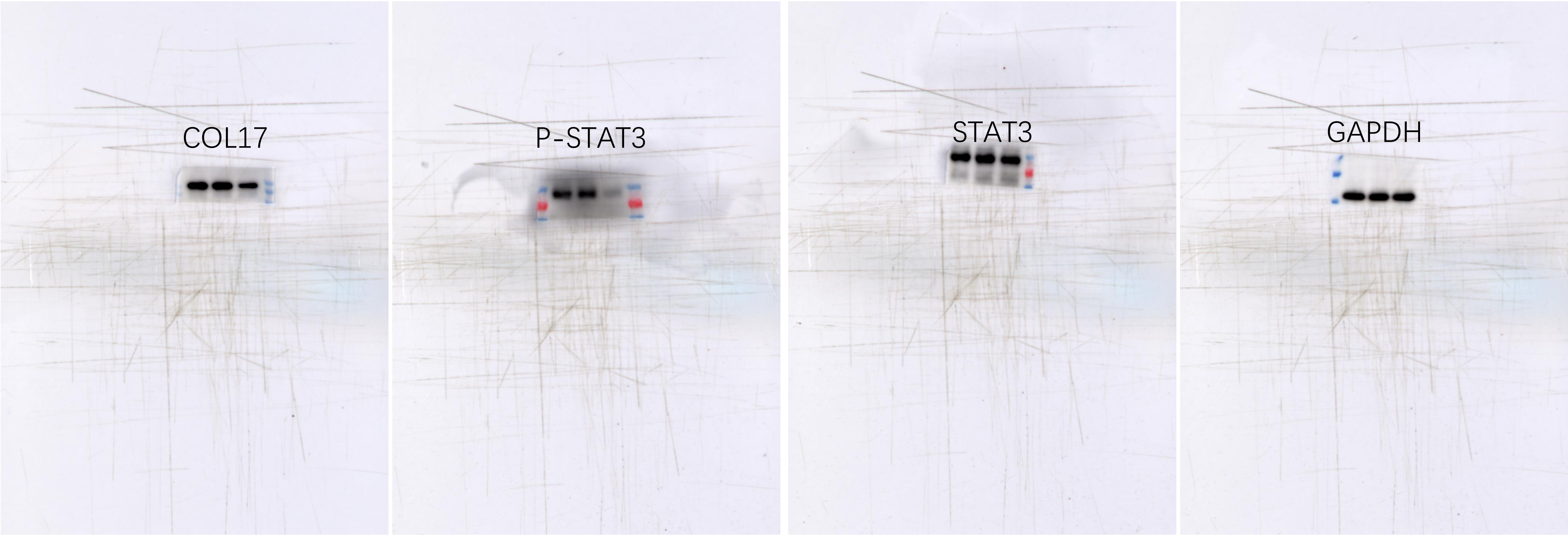

Fig 5A

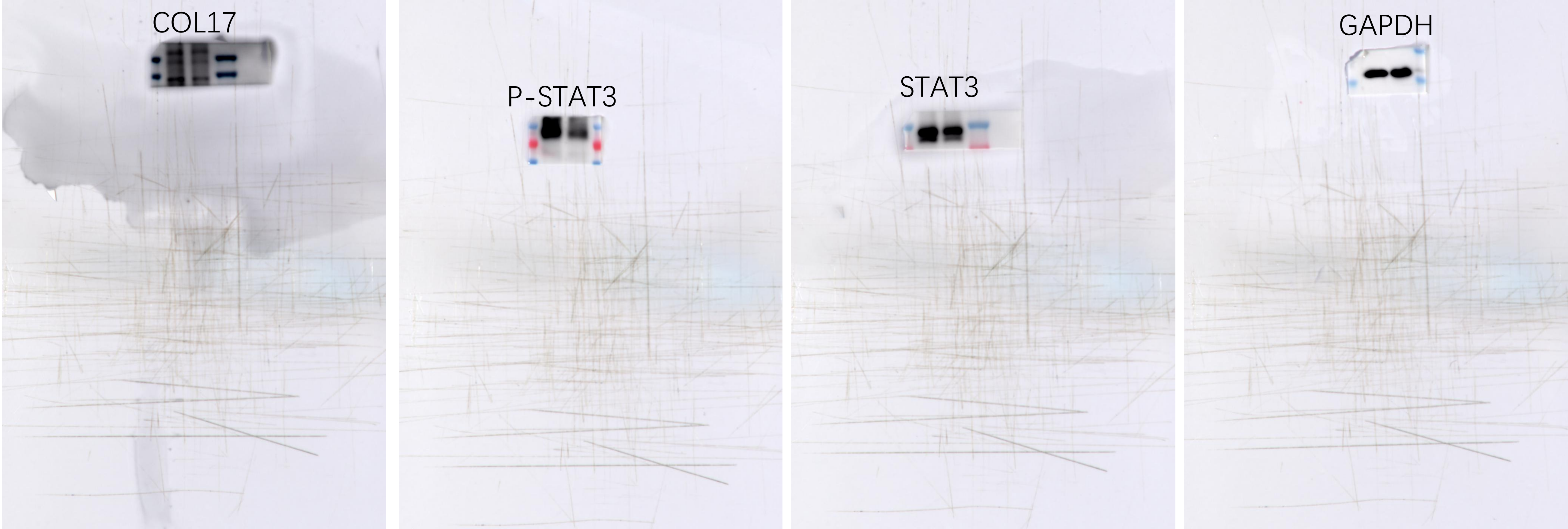

Fig 6A

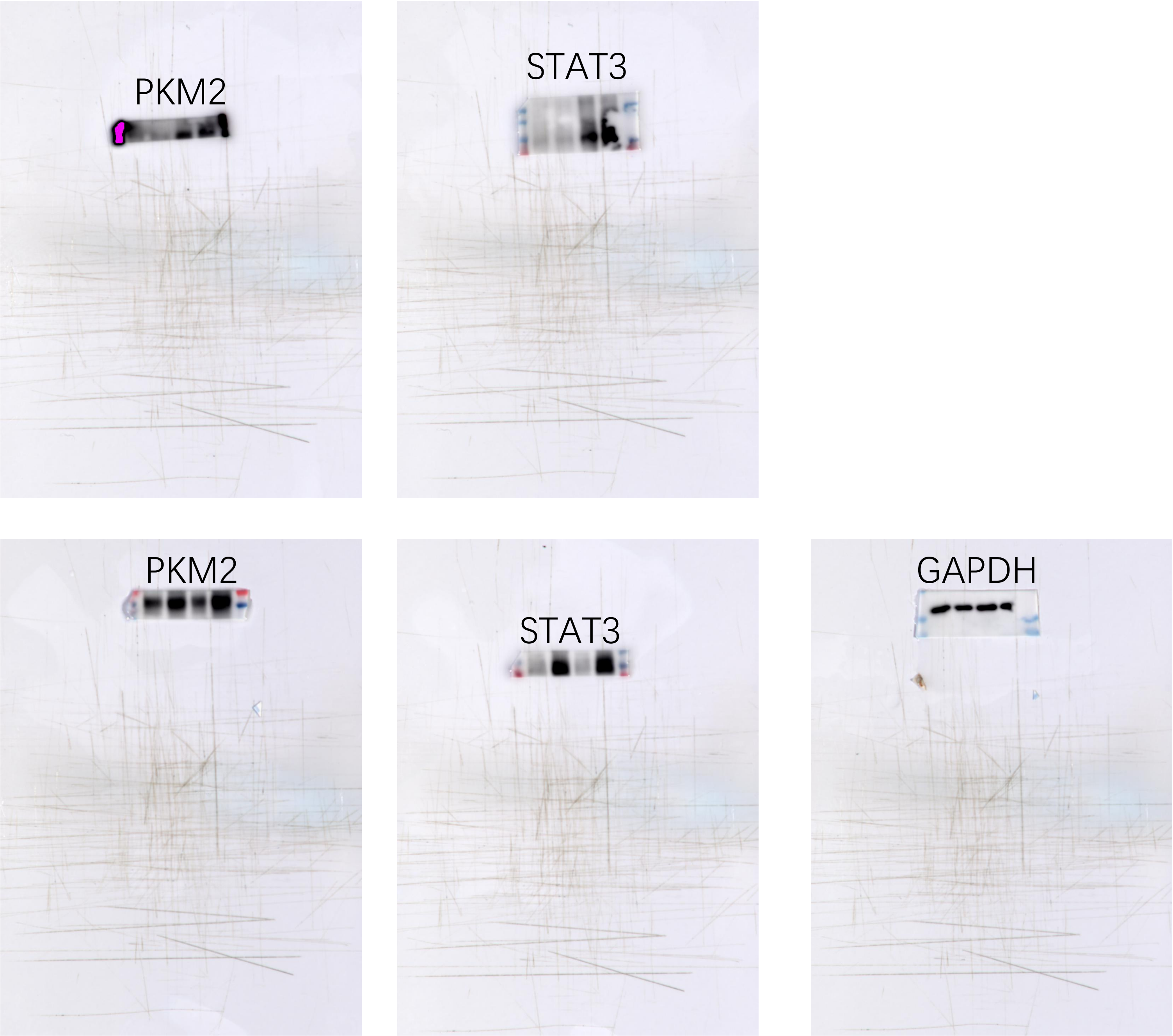

Fig 6A

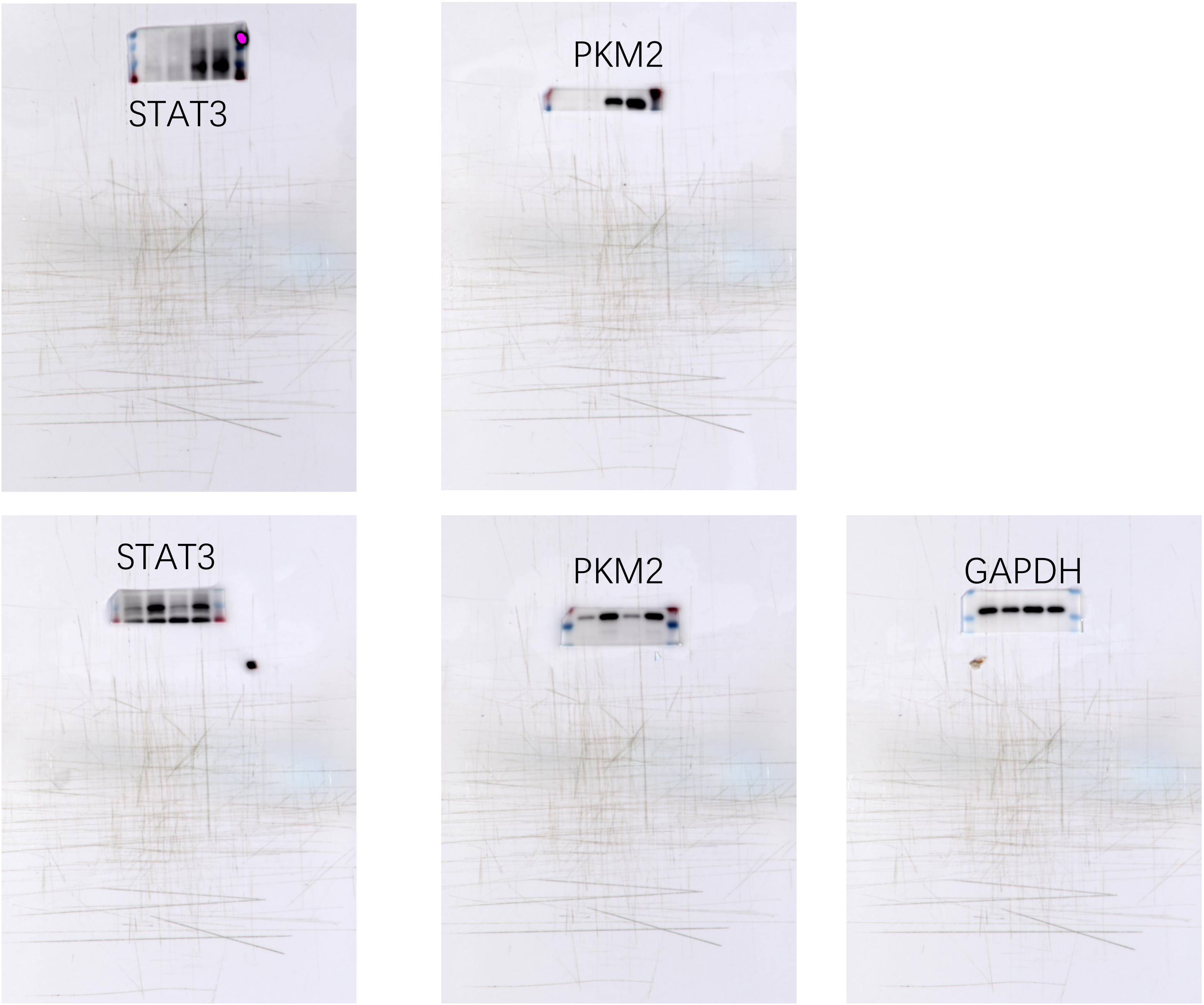

Fig 6B-total

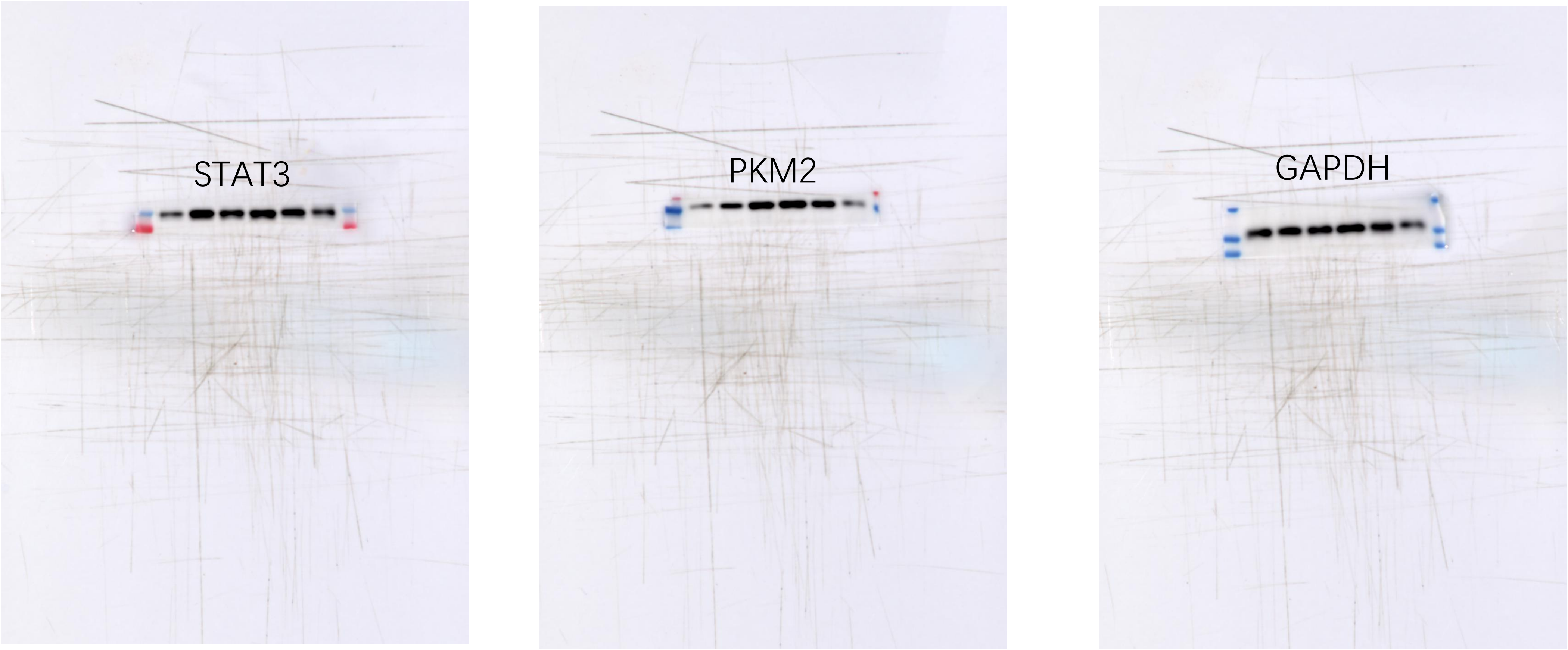

Fig 6B-cytoplasmic

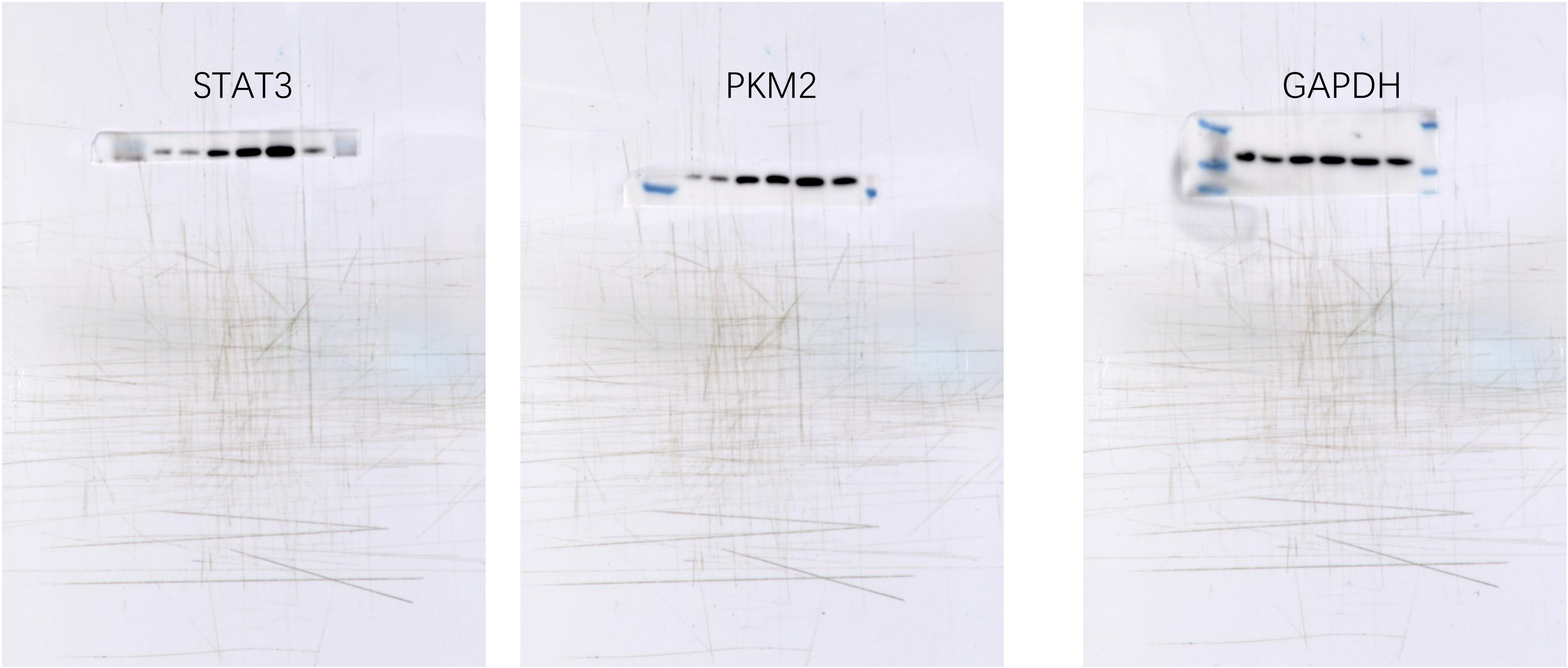

Fig 6B-nuclear

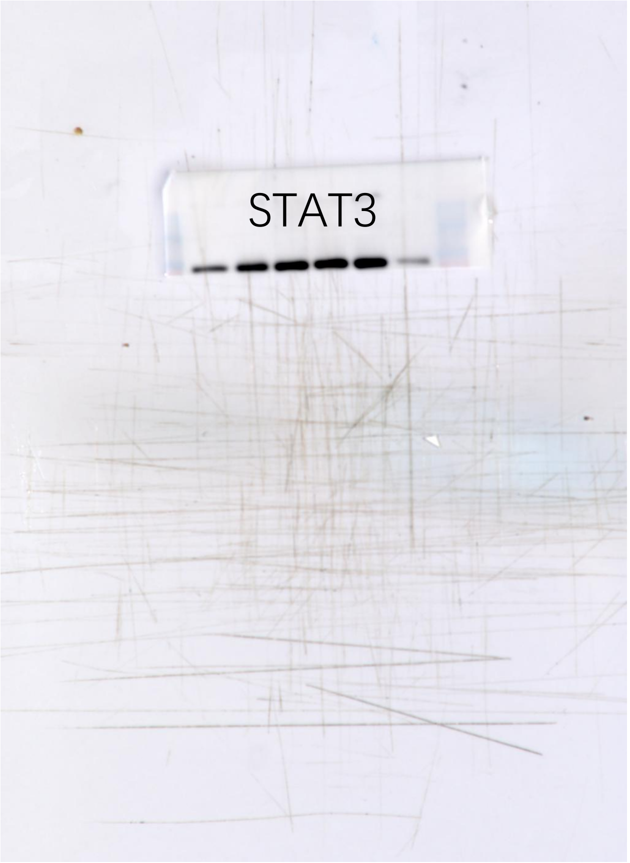

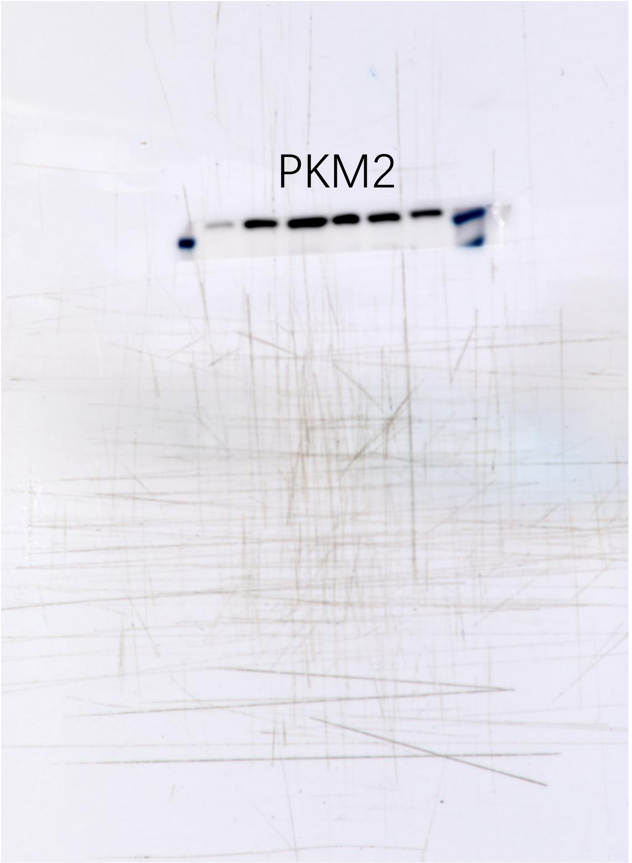

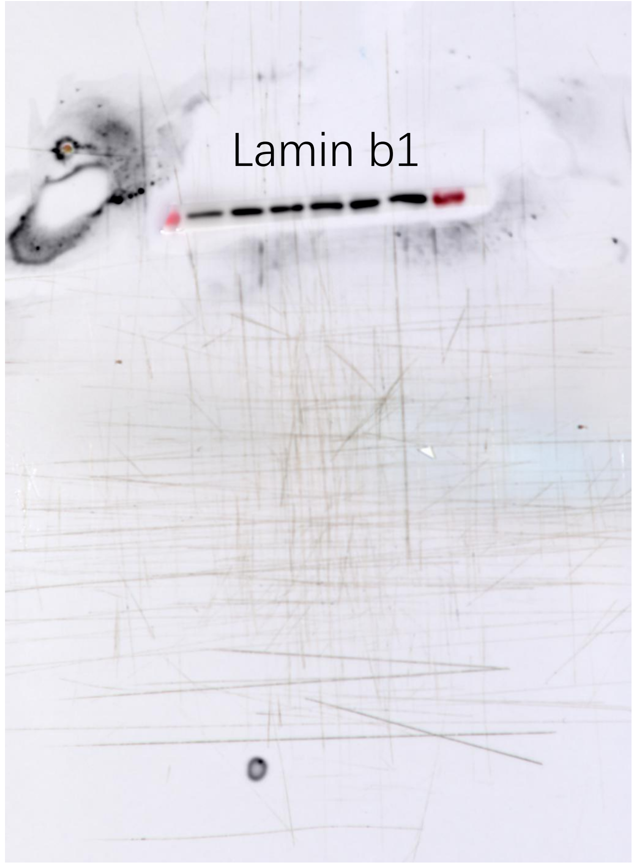

Fig 6C

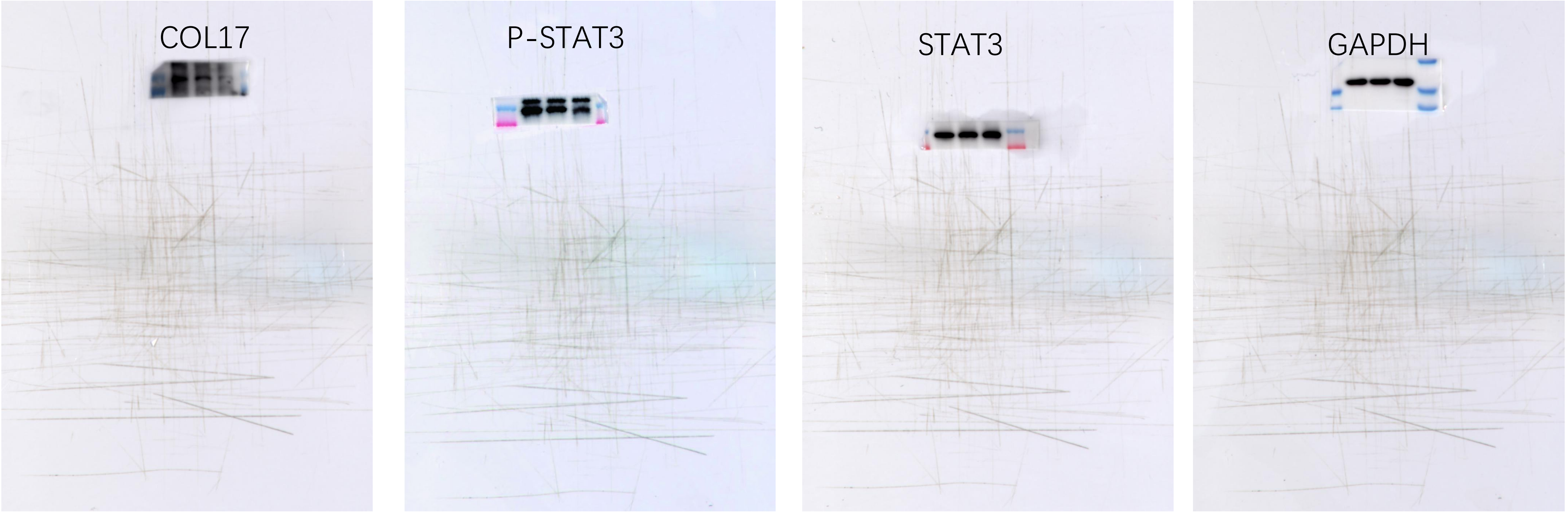

Fig 6D-nuclear

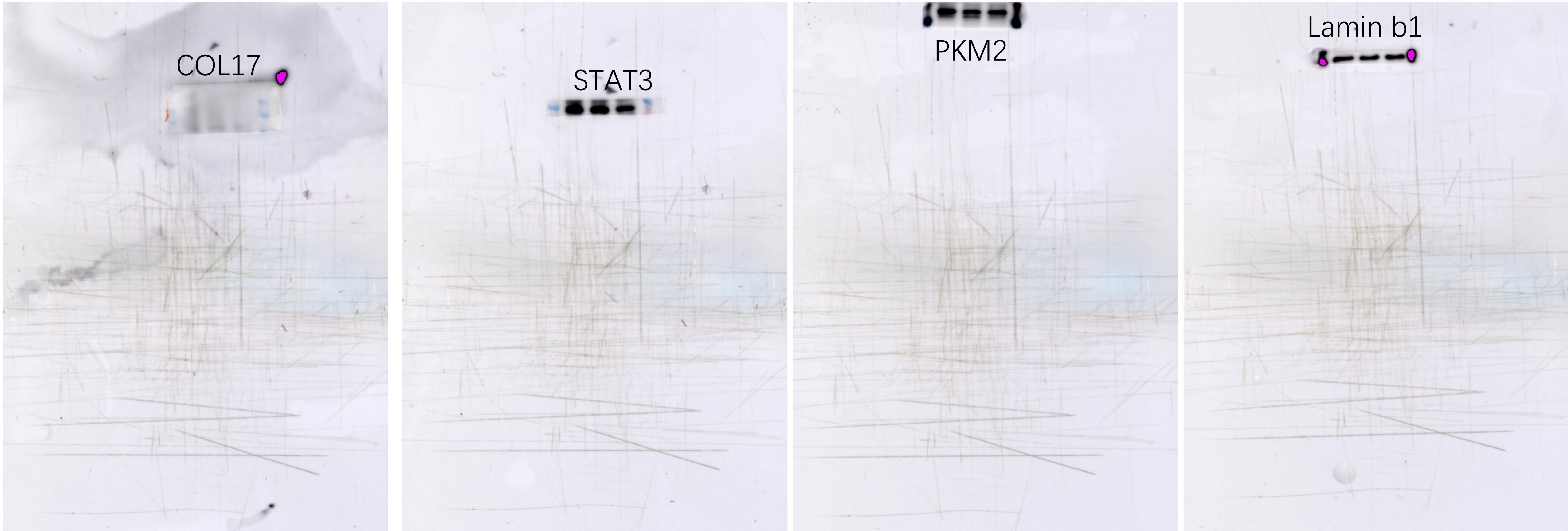

Fig 6D-cytoplasmic

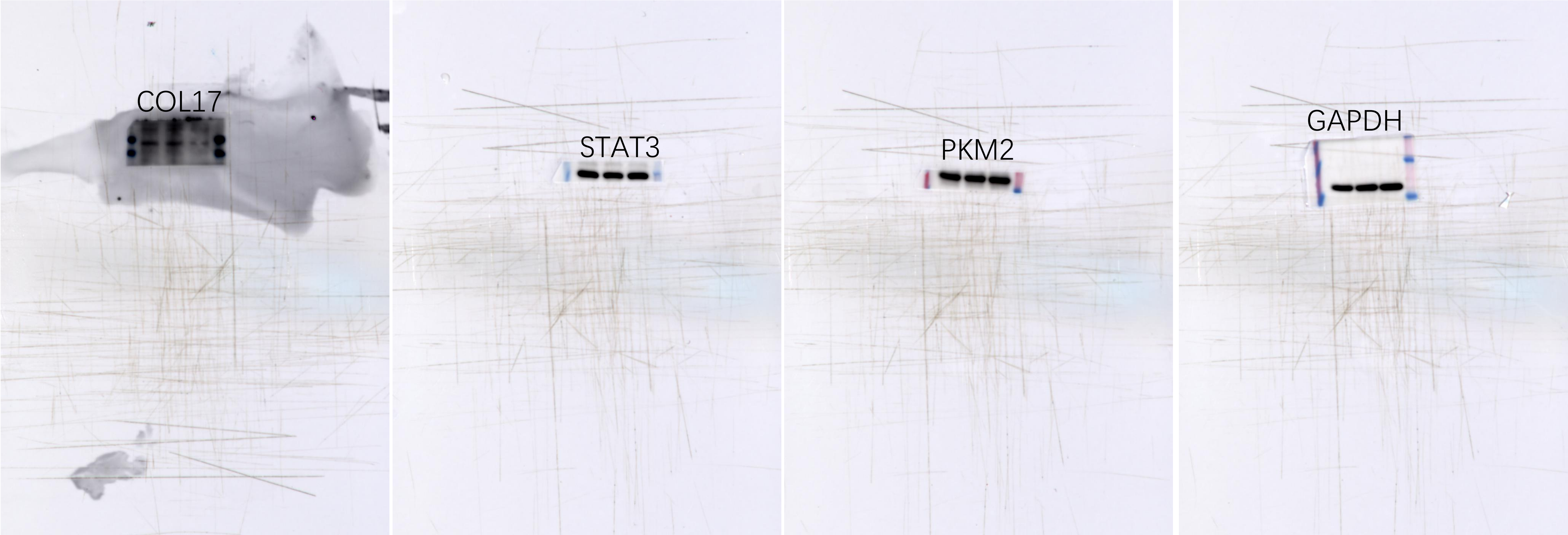

Fig 7A

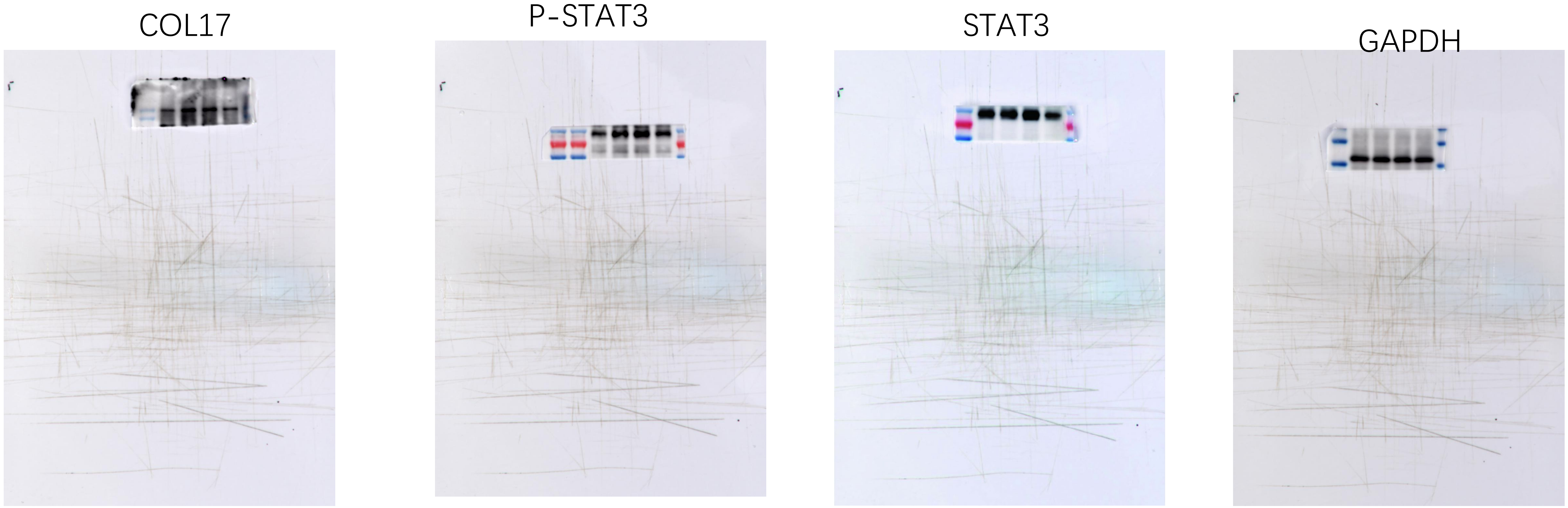

Fig 8A

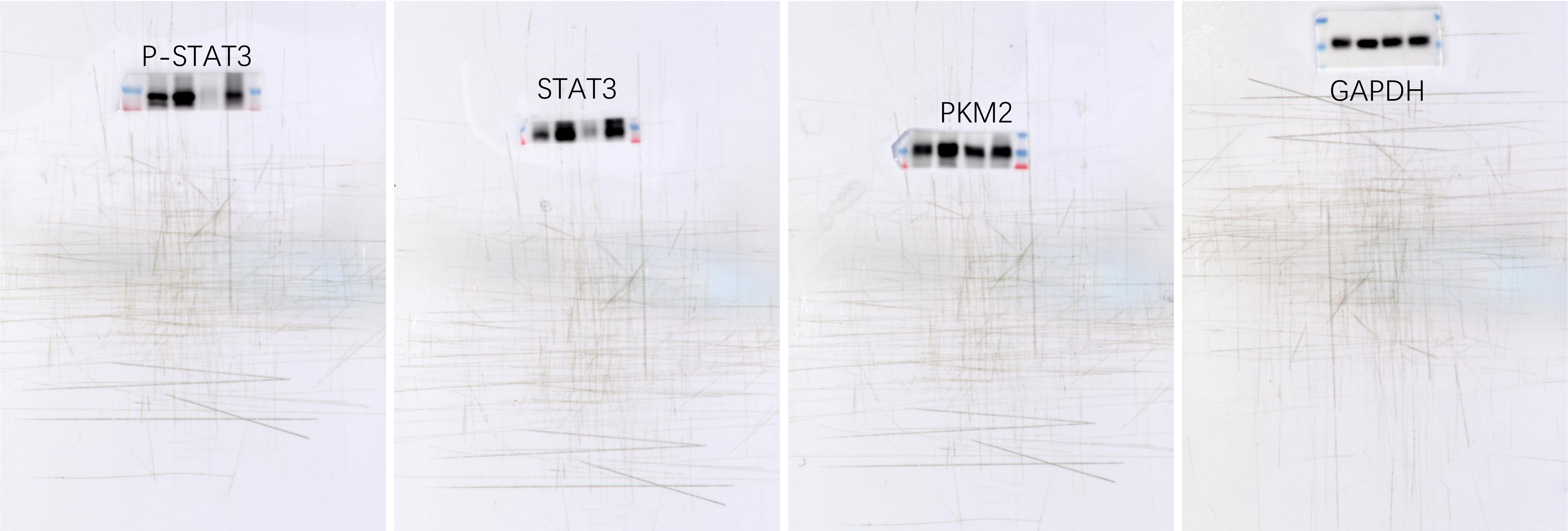

The membrane is up side down

Fig 8C

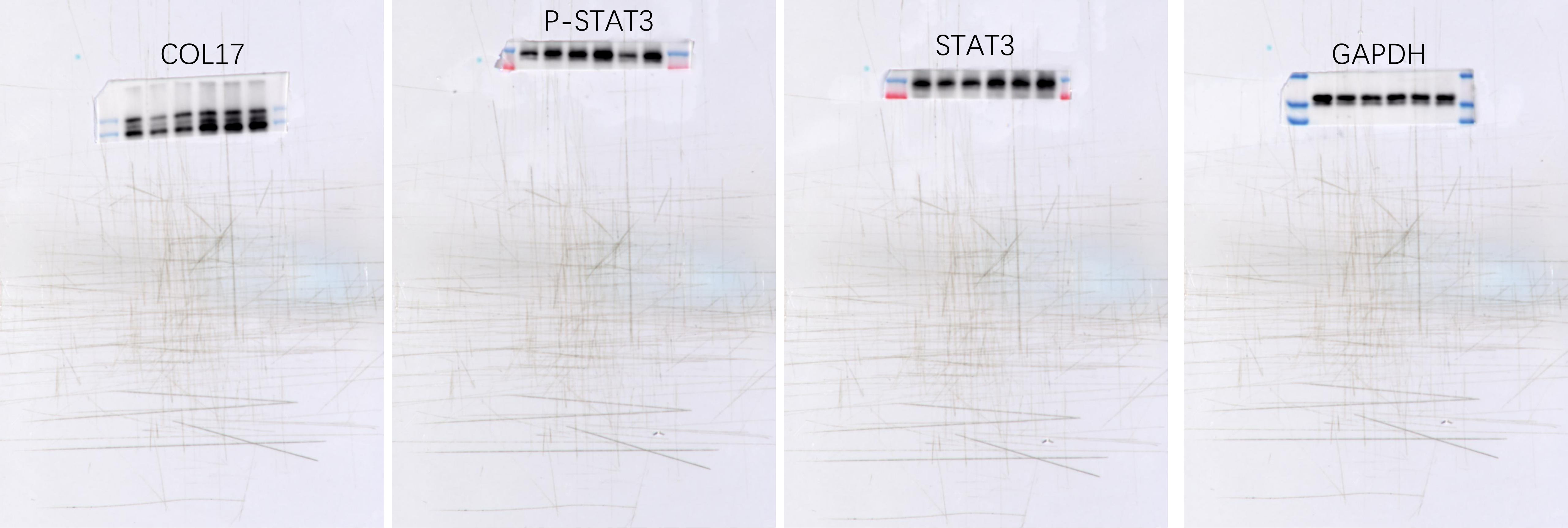

Supplement: Unedited blot and gel images [file jciinsight-10-184457-s286.pdf]
